# 3D spatial organization and network-guided comparison of mutation profiles in Glioblastoma reveals similarities across patients

**DOI:** 10.1371/journal.pcbi.1006789

**Published:** 2019-09-17

**Authors:** Cansu Dincer, Tugba Kaya, Ozlem Keskin, Attila Gursoy, Nurcan Tuncbag

**Affiliations:** 1 Department of Health Informatics, Graduate School of Informatics, METU, Ankara, Turkey; 2 Department of Chemical and Biological Engineering, Koc University, Istanbul, Turkey; 3 Research Center for Translational Medicine (KUTTAM), Koc University, Istanbul, Turkey; 4 Department of Computer Engineering, Koc University, Istanbul, Turkey; 5 Cancer Systems Biology Laboratory (CanSyL-METU), Ankara, Turkey; Queen's University, CANADA

## Abstract

Glioblastoma multiforme (GBM) is the most aggressive type of brain tumor. Molecular heterogeneity is a hallmark of GBM tumors that is a barrier in developing treatment strategies. In this study, we used the nonsynonymous mutations of GBM tumors deposited in The Cancer Genome Atlas (TCGA) and applied a systems level approach based on biophysical characteristics of mutations and their organization in patient-specific subnetworks to reduce inter-patient heterogeneity and to gain potential clinically relevant insights. Approximately 10% of the mutations are located in “patches” which are defined as the set of residues spatially in close proximity that are mutated across multiple patients. Grouping mutations as 3D patches reduces the heterogeneity across patients. There are multiple patches that are relatively small in oncogenes, whereas there are a small number of very large patches in tumor suppressors. Additionally, different patches in the same protein are often located at different domains that can mediate different functions. We stratified the patients into five groups based on their potentially affected pathways that are revealed from the patient-specific subnetworks. These subnetworks were constructed by integrating mutation profiles of the patients with the interactome data. Network-guided clustering showed significant association between the groups and patient survival (P-value = 0.0408). Also, each group carries a set of signature 3D mutation patches that affect predominant pathways. We integrated drug sensitivity data of GBM cell lines with the mutation patches and the patient groups to analyze the possible therapeutic outcome of these patches. We found that Pazopanib might be effective in Group 3 by targeting CSF1R. Additionally, inhibiting ATM that is a mediator of PTEN phosphorylation may be ineffective in Group 2. We believe that from mutations to networks and eventually to clinical and therapeutic data, this study provides a novel perspective in the network-guided precision medicine.

## Author summary

Precision medicine aims to find the best treatment strategy based on the information about the patient’s tumor. Molecular heterogeneity is the main obstacle in developing treatment strategies. Therefore, transforming patient specific molecular data into clinically interpretable knowledge is fundamental in precision medicine. In this work, we tackle the mutation profiles of patients with Glioblastoma Multiform (GBM) which is the most aggressive type of brain tumors with a poor survival. Our main motivation is that different mutations, that are spatially in close proximity in the same protein, or function in the same pathway, may result in phenotypically similar tumors. 3D spatial clustering of the mutations, that we call “mutation patch”, significantly decreases the heterogeneity. We additionally identify the affected patient-specific subnetworks and pathways that are inferred from mutations. Indeed, grouping the patients based on the presence of mutations in close proximity together with network-guided grouping is significantly associated with their survival. These results also enable us to suggest several therapeutic hypotheses for each group based on available drug treatment data. We believe that from mutations to networks and eventually to clinical and therapeutic data, this study provides a novel perspective to the analysis of mutation effects towards the network-guided precision medicine.

## Introduction

Cancer mostly occurs when somatic mutations accumulate and eventually change the behavior, structure and properties of the cell. Understanding which mutations cause cancer is of crucial importance. The large-scale cancer genome sequencing projects including The Cancer Genome Atlas (TCGA) [1], the International Cancer Genome Consortium (ICGC) [2], and smaller-scale gene/protein focused and genome-wide screenings have enabled us to explore a large volume of somatic mutations in human cancers. Heterogeneity in mutation profiles between and within tumors as well as among individuals of the same type of cancer is enormous. However, not every somatic mutation affects pathways involved in cancer. Mutations are conventionally divided into driver and passenger mutations based on their function in providing positive growth advantages to cancer cells. The main challenge is to discriminate the drivers from passengers. Lately, another class of mutations were defined which is called “latent” or “mini-driver” [3]. Latent mutations have a potential to behave like a driver or are not yet discovered to be as drivers. Although latent mutations are not significant mutations, they can be triggered to become driver mutations by the environmental factors or conformational changes in proteins. Ultimately, proteins of the driver genes are the favored molecular targets in drug discovery and cancer therapy. Also, having insights about the accumulation of mutations and their impact at the pathway level is equally important to understand the causes and mechanisms of cancer development and progression. All these together with epigenetic and post-translational factors determine the risk of cancer progression and the therapeutic resistance. One therapy that works in some patients might be ineffective in other patients. It is challenging even in a single patient for the same tumor type.

Protein-protein interactions (PPIs) have critical role in regulating and performing many cellular functions. Disease-associated mutations are more likely to affect protein interactions and eventually the cellular functions [4]. Several studies have focused on the impact of the disease-associated alterations in protein-protein interaction networks [5–8]. Recently, IMEX consortium [9] started an effort to curate and catalogue the oncogenic and neutral mutations in protein interactions [10]. The combination of three-dimensional structural information with large-scale mutation information may assist clarifying the impacts of cancer mutations [7, 11–14] as a protein's biological functions and physical interactions are strongly linked to its structure. Various mutations in the same protein may result in distinct profiles of interaction and eventually distinct phenotypes of disease [15–19]. Mutations that destabilize a protein’s global structure can result in severe alterations in its overall interactions. Additionally, a mutation may affect only one interface of a multi-face protein and the lost and gained interaction partners through the affected site may give insights about the functional changes. This type of edgetic perturbations [4] in proteins thus require structurally resolved PPI networks and the 3D spatial position of the mutations in proteins [20–22]. Mutations in cancer have been evaluated in many studies based on their organization in proteins structures [5, 6, 23, 24]. Niu et al spatially clustered the mutations from 19 different cancer-types and came up with the set of druggable functional mutations [24]. The functional effects of mutations on protein interactions and signaling networks have been extensively reviewed in [14] which nicely puts forward that biophysical studies complement omics and clinical data. Additionally, some other studies focused on patient-specific analysis of the molecular signatures in tumors in a network context [25–27]. The phosphoproteomic data from eight GBM patients have been previously used to demonstrate that the network-guided comparison reveals commonalities and differences across patients [27]. In another network-based approach, mutations, transcriptional and phosphoproteomic data were used to model patient-specific pathways in prostate cancer [25]. The network based stratification (NBS) approach integrated somatic mutation profiles with molecular interactions to divide a heterogeneous set of tumors into clinically similar clusters [28] which was successfully applied to various TCGA mutation profiles [28, 29]. NBS was earlier used in conjunction with structural locations of cancer missense mutations to disclose the impacts of a mutation in the core or interface regions when the rebuilt networks are perturbed [30]. Network-based analysis was further used to distinguish driver mutations from passenger mutations in GBM [31].

**Fig 1:**
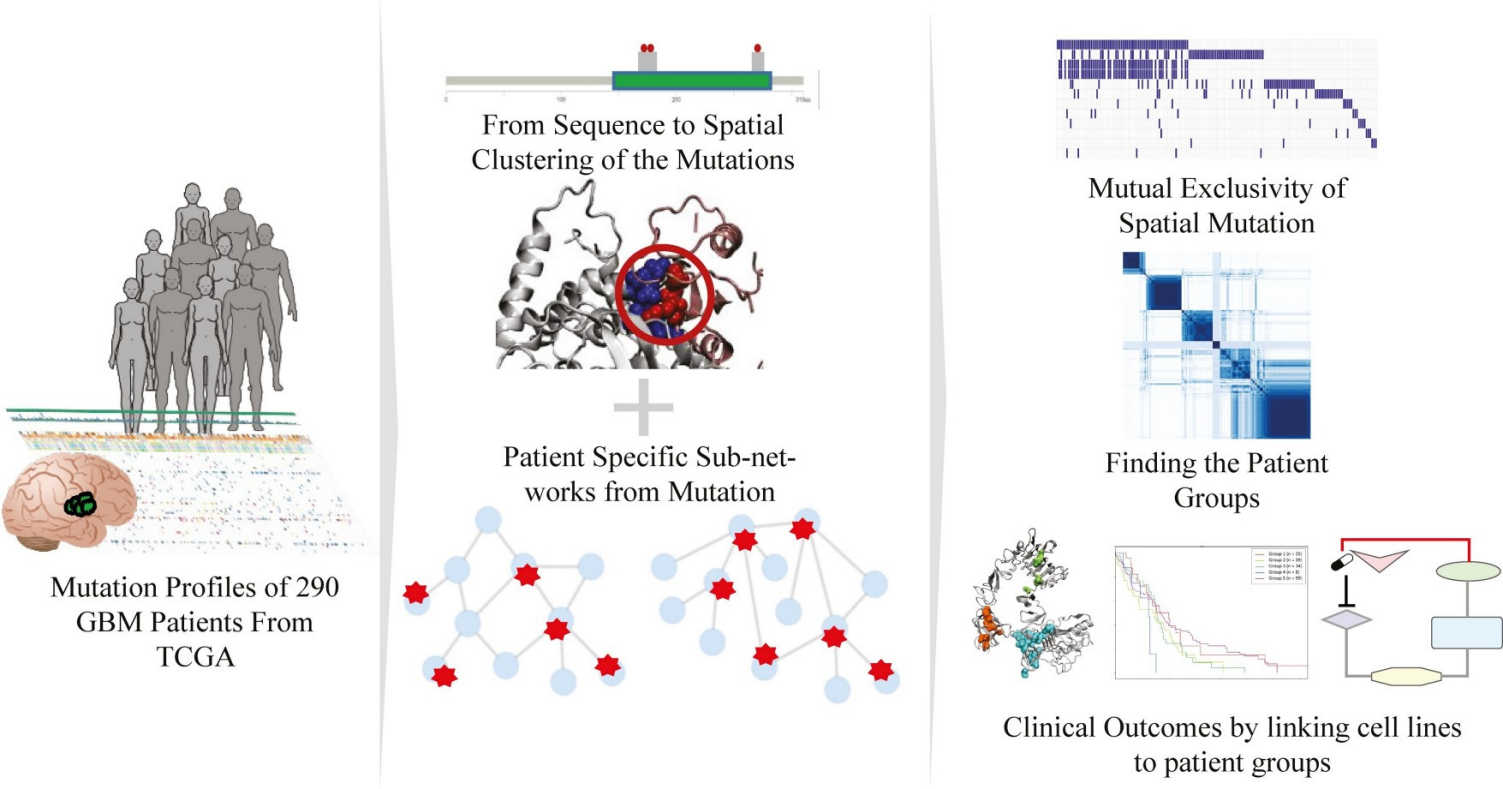
Overview of the method. Patient specific GBM tumor mutation profiles were obtained from TCGA. The spatial proximity of each mutation is searched and mutation patches were obtained. Simultaneously, each cancer related driver protein having at least one mutation in each patient was used to reconstruct patient-specific sub-networks. Red dots and stars in the middle panel correspond to mutations mapped to the sequence, structure and PPI network. Finally, the sub-networks were used to classify the patients, to find signature patches in each patient groups and to demonstrate the help of 3D patches in overcoming heterogeneity. Lastly, we investigated the patient groups to find an association with the clinical outcome by using cell line drug sensitivity data. The brain and human icons in the first panel are retrieved from Reactome Icon Library [32].

Computational approaches are crucial for analyzing the effects of mutations on proteins, protein interactions and functional pathways in a patient-specific way, considering the big quantity of diverse data including mutations, protein structures, and known PPIs. We applied a systems level approach to the somatic missense, nonsense and frameshift mutations in 290 Glioblastoma (GBM) patients which is the most aggressive type of brain tumor. The mutation profiles are rarely common across the patients and they do not track with the known transcriptional subtypes of GBMs or the known biomarkers such as the IDH1 mutation. Despite this heterogeneity, mutations in different proteins functioning in the same pathway may result in phenotypically similar tumors. In order to overcome the heterogeneity in tumors and develop personalized therapeutic strategies, reverse engineering from mutations to networks and pathways is a key approach. In this work, we proceeded in two directions: (i) finding the spatial arrangement of the mutations as patches and (ii) reconstructing the sub-networks primarily affected by the set of mutations across patients (see Fig 1). Then, each patient-specific network was reduced into a significantly enriched set of pathways and patients were grouped to better classify them into clinically similar groups according to their pathway similarity. Toward the precision medicine, patient groups were analyzed based on their predominant patches and pathways, and were associated with their survival profiles. Eventually, drug sensitivity data in GBM cell lines were integrated with the signatures of patient groups and hypothetical therapeutic strategies for each patient group were inferred.

## Results

### Heterogeneity decreases with the 3D grouping of GBM mutations

The missense, nonsense and frameshift mutations from 290 GBM tumors were first analyzed at the sequence level. There are 15,399 unique mutations and 14,308 of them match at least to one canonical protein whereas the rest matches to alternative isoforms of the proteins. The average number of mutations per patient is 50.43. The mutations are rarely common across different GBM tumors where only 44 mutations are present in at least three patients and 213 mutations are present in at least two patients. The most frequent mutations with 13 patients are EGFR mutation A289V and IDH1 mutation R132H.

Next, we mapped the GBM mutations on to protein structures and found that 4702 mutations were aligned to at least one protein structure either from PDB [33] or from ModBase [34]. The local organization of mutations in 3D was determined by their spatial proximity to each other which we call “patches”. A patch is a set of mutated residues that are either in physical contact with another mutated residue (that is, at least one pair of atoms within 5Å distance), or there is another intermediate residue in close proximity connecting the two mutated residues. The term “patch” was used in previous studies, however we have to indicate that our patch definition is different from those [11, 24]. We looked for continuous residue contacts instead of using a mutated residue as the center of the patch. The 3D spatial grouping of 4702 mutations resulted in 220 patches composed of 580 mutations and 4122 singletons (a mutation that is not involved in a patch). We then split patches as intra- and inter- which represents patches that do not include any interface mutations and patches that have at least one interface mutation, respectively. The interpatch can consist of residues of a single protein or two partner proteins. In total, there are 160 intra-, 60 inter-patches in our dataset.

**Fig 2:**
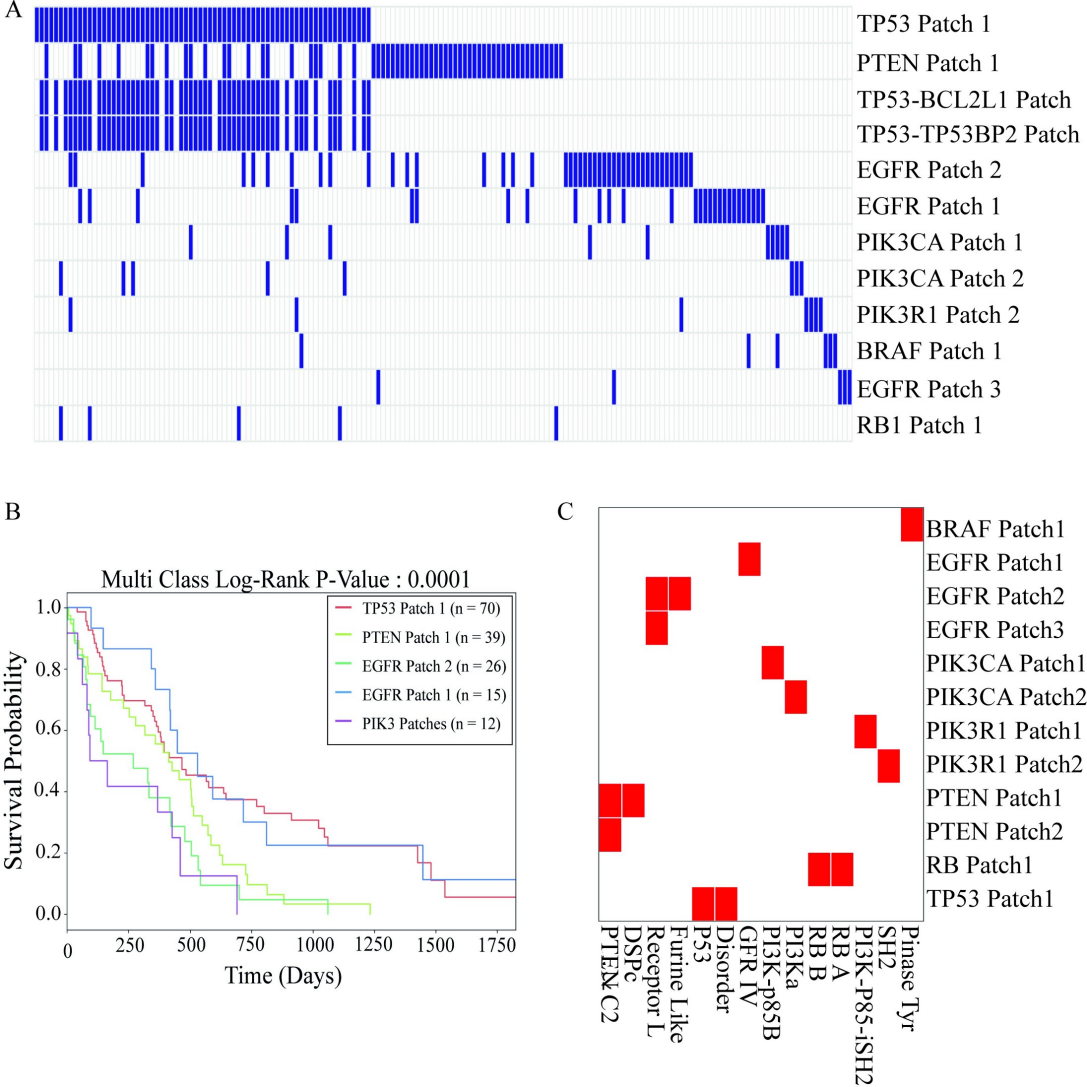
The 3D patch profile and survival curve of the patients having at least one patch mutation. (A) Grouping patients based on 3D patches. Each column represents a patient and each row represents patches that are present in at least 2% of patients. (B) Kaplan-Meier survival curves of the patient groups. (C) Mapping patches of frequently mutated hub proteins to their domains. Red colors represent the presence of patches in the corresponding domains.

A patch is present in a patient if the patient has at least one patch mutation. While each individual mutation is shared between 1.13 patients on average, each patch is present in 3.5 patients on average, which partially reveals some common patterns across patients. In Fig 2A, patches are sorted based on their frequency across patients. The patches in TP53 and PTEN are common among 20% of the patients, and the patches in EGFR are common in 8% of the patients, that yields slightly better detection of commonalities across patients.

Then, we divided the patients into mutually exclusive groups based on their most common patches. We linked patient survival data to assess the advantage of patches (3D spatial grouping) in overcoming heterogeneity. The 3D spatial grouping of patients is significantly associated with survival data (P-value = 0.0001 for 162 patients having at least one mutation in patches). We found no significant association of individual mutations between survival and patient groups. (P-value = 0.5115- S1 Fig). Previous studies suggested that 3D clustering of the mutations led to a better classification of different cancer types, driver mutations or novel cancer genes [5, 11, 24, 35]. Our initial results suggest that patient-grouping is also possible with 3D patches. The strong association between the patient groups and their survival indicates that patients with similar 3D spatial organization in their proteins may have similar disease phenotypes which may represent similar affected functions and pathways in the tumor cells.

In order to understand the possible functional effects of spatial organization, we mapped the patches on protein domains. We found that different patches are located in different functional protein domains. For example, PIK3R1 gene encodes the P85 which have two patches: Patch1 is on the inter-SH2 domain that has the inhibitory function on PIK3CA by binding the catalytic domain p110, on the other hand Patch2 is on the SH2 domain where the protein binds to phosphorylated residues. Additionally, PTEN has two patches and one patch is on the phosphatase domain, the other on membrane binding domain of the protein. Mutations on PTEN Patch1 disrupts the phosphatase function which results in accumulation of PIP3 [36, 37] in cell and thereby activation of the AKT pathway that leads to tumor growth. PIK3CA protein consists of four different domains where Patch1 is only in the region in which the catalytic domain of PIK3CA, p110 binds the P85 subunit which is the p110 inhibitor. In Fig 2C, we showed the domains where the patches are mainly located.

**Fig 3:**
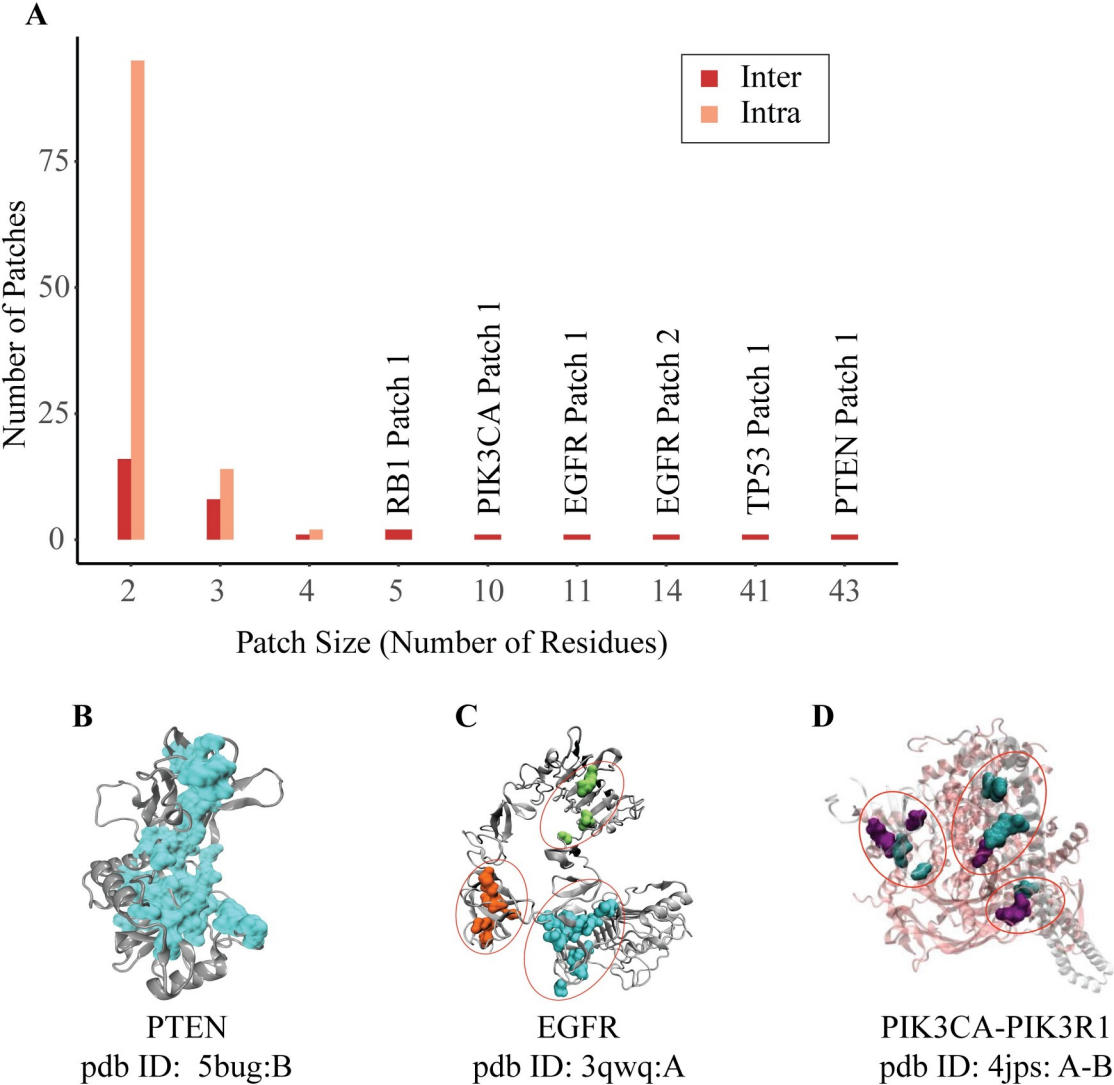
(A) Histogram of the patch sizes for intra- and inter- patches. While frequently mutated hub proteins have relatively large patches, most patches are small in size. (B) PTEN Patch 1—patch size—43 residues—an example of mutations forming a residue network from the surface to the core. (C) EGFR—three patches with different sizes: 11, 14 and 5 residues, respectively. (D) PIK3R1-PIK3CA—three inter-patches—each patch has at least one mutation in each partner protein.

So far, our analysis did not differentiate driver mutations from passenger mutations. Driver genes can, when mutated, play a causal role in tumorigenesis and should be enriched for driver mutations. In our analysis, we wanted to eliminate the noise from passenger mutations, therefore a list of putative driver genes was assembled from various literature sources and databases including The Network of Cancer Genes [38], Cancer Genome Interpreter [39], COSMIC [40] and the Firehose data using CHASM [41], MutSig [42] and Mutations Assessor [43] for GBM and the analysis focused on those genes. In total, we obtained 6270 driver mutations from 3789 driver genes. We then found the intersection between the TCGA GBM mutation dataset and the collected driver genes and driver mutations. Of all the mapped mutations, 6278 are located in a driver gene of which 2072 mutations map to at least one protein structure. When we filtered out our 3D patch dataset based on the driver genes, we obtained 112 intra-, 32 inter-patches. There are numerous patches which only contain two or three residues (Fig 3A). These small patches tend to be intra-patches without any interface mutations. On the other hand, the larger patches happen to be inter-patches (with at least one interface residue) and the largest ones are found in the central proteins (TP53 with 41, PTEN with 43 residues as shown in Fig 3A). An example of a large patch in PTEN is illustrated in Fig 3B. Some proteins have various patches of comparatively small size such as EGFR (its three patches are shown in Fig 3C). An example of inter-patches is in the PIK3R1-PIK3CA complex where both partner proteins have at least one mutation (Fig 3D).

We further compared the differences in 3D spatial organization of their mutations between tumor suppressors and oncogenes. Interestingly, we found that driver mutations of the tumor suppressors have a tendency to be located in patches whereas driver mutations of the oncogenes mostly remain as singletons (P-value = 8.33x10^-6^/ Fisher’s Exact Test). These results may explain the reason why PTEN (43 and 2 residues in two different patches) and TP53 (41 residues in only one patch), which are tumor suppressors, have relatively larger patches. On the other hand, PIK3CA (10, 3, 2, 2 residues in four different patches of PIK3CA) and EGFR (11, 14, 5 residues in three different patches) oncogenes have smaller patches and also many singletons. These results agree that it is more difficult to make a protein more active or efficient, therefore mutations in oncogenes tend to pile up at very specific sites, i.e. all cancer-related Ras mutations are around the GTP binding site. Whereas tumor suppressors can be functionally impaired in a variety of ways and thus mutations could be more broadly distributed in large patches. Additionally, we further compared oncogenes and tumor suppressors based on the frequency of non-synonymous mutations and found that frameshift and nonsense mutations are significantly more frequent in tumor suppressors (P-value = 1.84x10^-15^). These types of mutations may disrupt functionality of tumor suppressors making cells more vulnerable to cancer.

#### Structural mapping of GBM mutations and physicochemical organization in proteins and protein interactions

From the chemical and structural perspectives, we evaluated the characteristics of GBM mutations at the molecular level. Mapping mutations onto protein structures enabled us to find their locations in proteins. Knowledge of the locations and physicochemical properties of mutated residues enhances our understanding of their functional impact in the cell. Therefore, we divided the mutations into three classes based on their location in protein structure, namely, interface (physically contacting to a partner protein), core (no solvent accessibility) and surface (having solvent accessibility excluding interface residues) mutations. Most of the mutations are located in the surface region of these proteins (see Table 1 for the summary of all numbers). Binding site information was retrieved from PDB (if there exist complex structures), or from models of Interactome3D [7], Interactome Insider [12], and PRISM [44] as shown in Table 1. While most mutations are found as singletons, approximately 10% of the mutations are in close proximity to each other and form spatial patches. Additionally, 121 of 757 interface mutations are located in patches (~16% of all interface mutations). Therefore, interface mutations are more populated in patches compared to non-interface mutations (odds ratio = 2.09, Fisher’s Exact Test P-value < 0.0001).

**Table 1:** Number of mutations mapped to protein structural regions.

Number of mutations from TCGA: 15399			
Structural Region	All	PDB	Models
Core	861	372	ModBase: 489
Surface	3084	1153	ModBase: 1931
Interface	757	340	Interactome3D: 74 PRISM: 60 ECLAIR: 283
Total	4702	1865	2837

**Fig 4:**
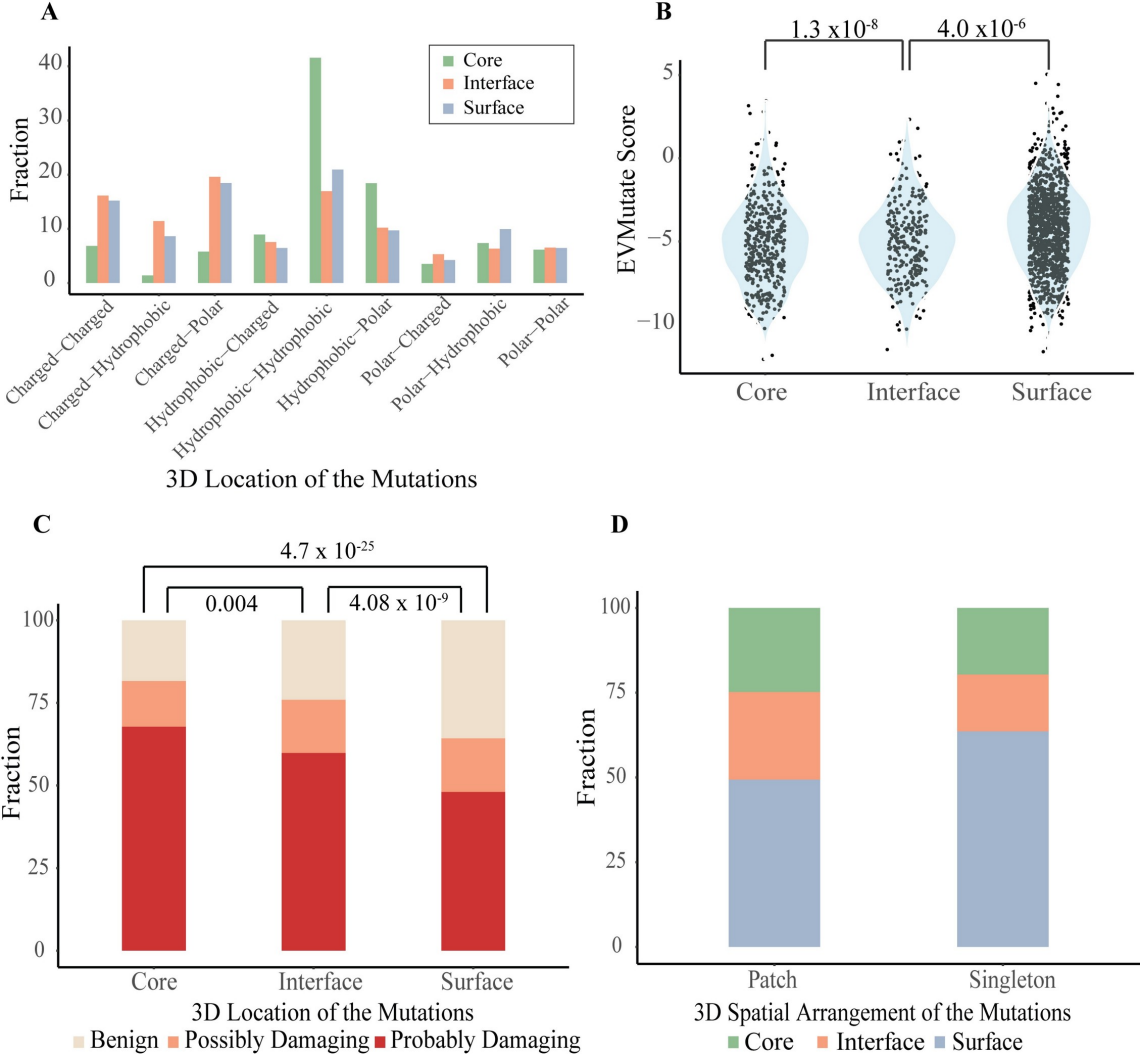
The characteristics of the mutations. (A) Changes in the chemical properties of the driver protein mutations according to their physical locations (B) Distribution of mutations according to their disease association (EVmutation score) in different locations. The more negative EVmutation score implies the more damaging mutation. (C) Fraction of mutations according to their disease association (PolyPhen-2 status: benign, possibly damaging, probably damaging) in different locations. (D) Fraction of patch mutations and singletons according to their locations.

We analyzed whether a mutated residue on a driver protein preserves its wild type chemical class or switches to another chemical class. (Fig 4A). Chemical classes were defined as hydrophobic, charged and polar. Most core mutations are hydrophobic and their chemical classes are preserved. Proteins with a large core region are generally robust to the effect of mutations. [45]. However, compared to the surface region, the core region is relatively less robust to non-hydrophobic alterations. Substitution to polar residues, for instance, significantly affects protein packing and folding. [46, 47]. Therefore, the set of mutations that change their chemical class in the core region is expected to have significant functional effects. Our analysis shows that chemical class profiles of interface and surface mutations are very similar to each other and substantially different from core mutations. Surface and interface residues are more likely to change their chemical class (P-value = 1.81x10^-11^). When we focused only on the driver gene mutations, the results did not change. The most prominent changes in surface and interface mutations are charged-to-charged and charged-to-polar shifts. Other shifts, such as changes from hydrophobic-to-polar and from hydrophobic-to-charged, are less frequent. Although the fraction of hydrophobic-to-hydrophobic is also high in interface and surface regions, this is less than the expected fraction according to the Chi-square test (P-value = 2.29x10^-42^). Nishi et al. found that GBM missense mutations on protein-protein interfaces have overall destabilizing effect and mostly alter the electrostatic component of binding energy [13]. They also showed that mutations on interfaces resulted in more drastic changes in physicochemical properties of amino acids than mutations that are located outside the interfaces. David and Sternberg showed that there are differences in polarity, hydrophobicity and charge changes between polymorphisms and disease causing mutations [48]. The distribution of physicochemical changes was significantly different between disease-causing SAVs and polymorphisms and in different protein regions. We also found similar results that the most frequent changes are in charged interface residues. Together with the chemical class shifts in the core region, this kind of changes are expected to be functionally critical and alter the protein binding or solubility characteristics.

Another interesting observation is that some positions are mutated differently in patients. There are 70 unique positions representing such cases. For example, Proline appeared to be mutated to Arginine at 596th position of EGFR in one patient while it is mutated to Leucine or Serine in other patients. We also checked their chemical properties and found that 63% of all these 70 positions were originally charged amino acids. The most frequent alterations in these positions are either preserving charged class or changing from charged to polar. These are mostly found in interfaces and surfaces. Alterations to hydrophobic amino acids are very rare in these positions.

#### 3D mutation patches and disease association

The impact of mutations on the proteins and their interactions is not uniform. Therefore, we further assessed the effect of mutations on their disease-causing potential using two different methods and analyzed those effects according to the location of the mutations. The first, EVmutation [49] utilizes an unsupervised statistical method that considers mutations with co-evolution of the neighboring residues while the second, PolyPhen-2 [50] uses a learning-based approach that incorporates sequence- and structure-based features such as sequence conservation, domain information, buried surface area. Both methods are based on multi-cellular phenotypes such as disease association or evolutionary conservation to classify the mutations as damaging or neutral. The more negative the value of the EVmutation score the more damaging is the mutation. PolyPhen-2 on the other hand classifies mutations as benign, possibly damaging and probably damaging. Although these methods are not trained on cancer mutations, they still classify the mutations on driver genes as more damaging compared to the mutations on passenger genes (P-value according to EVMutation = 6 x10^-44^, P-value according to Polyphen2 = 2 x10^-64^). Additionally, we found that mutations on tumor suppressor genes are slightly more damaging than oncogenes (P-value = 0.015). Both methods gave similar results that core and interface mutations are more damaging compared to surface mutations (Fig 4B and 4C), whereas the damage is more severe in the core region based on PolyPhen2 (P-value = 0.004). The results also show that interface mutations in patches are more damaging compared to singleton ones (P-value = 0.002). Similar results were also obtained from EVmutation analysis.

We obtained PolyPhen-2 results for 703 interface mutations of which 487 are located on driver genes as detailed in Table 2. 81% and 93% of singletons and patch mutations are damaging (possibly or probably), respectively. The impact of mutations in hub proteins and the rest, however, is distinct from each other. We note that hub proteins are frequently mutated while other proteins have rare mutations. We observed that patch mutations are more damaging in hub proteins (PTEN, TP53, EGFR, PIK3CA, RB1 and PIK3R1, P-value = 0.00028/Chi Square Test). However, singletons are more damaging in the rest (P-value = 0.00029/Chi Square Test). We showed in a previous study that energetically important hot spots can be found as singlets or clustered in hot regions. Most of the disease causing single amino acid variations in that dataset -restricted to human proteins only but not limited to cancer variations- were found as singletons rather than in hot-regions [17]. Compared to the hot spot organization of the disease variations, our new results add another layer of information (hub proteins vs. the rest) that cancer mutations in interfaces of hub proteins are frequently located in 3D patches while interface mutations in other proteins stay as singletons.

**Table 2:** Disease association of singleton and patch mutations in the interface region of the hubs and the rest.

	Frequently mutated proteins		Rest		Total	
	Patch	Singleton	Patch	Singleton	Patch	Singleton
Benign	1	5	7	67	8	72
Possibly Damaging	11	0	2	63	13	63
Probably Damaging	69	1	21	240	90	241
Total	81	6	30	370	111	376

#### Characteristics of interface mutations

**Fig 5:**
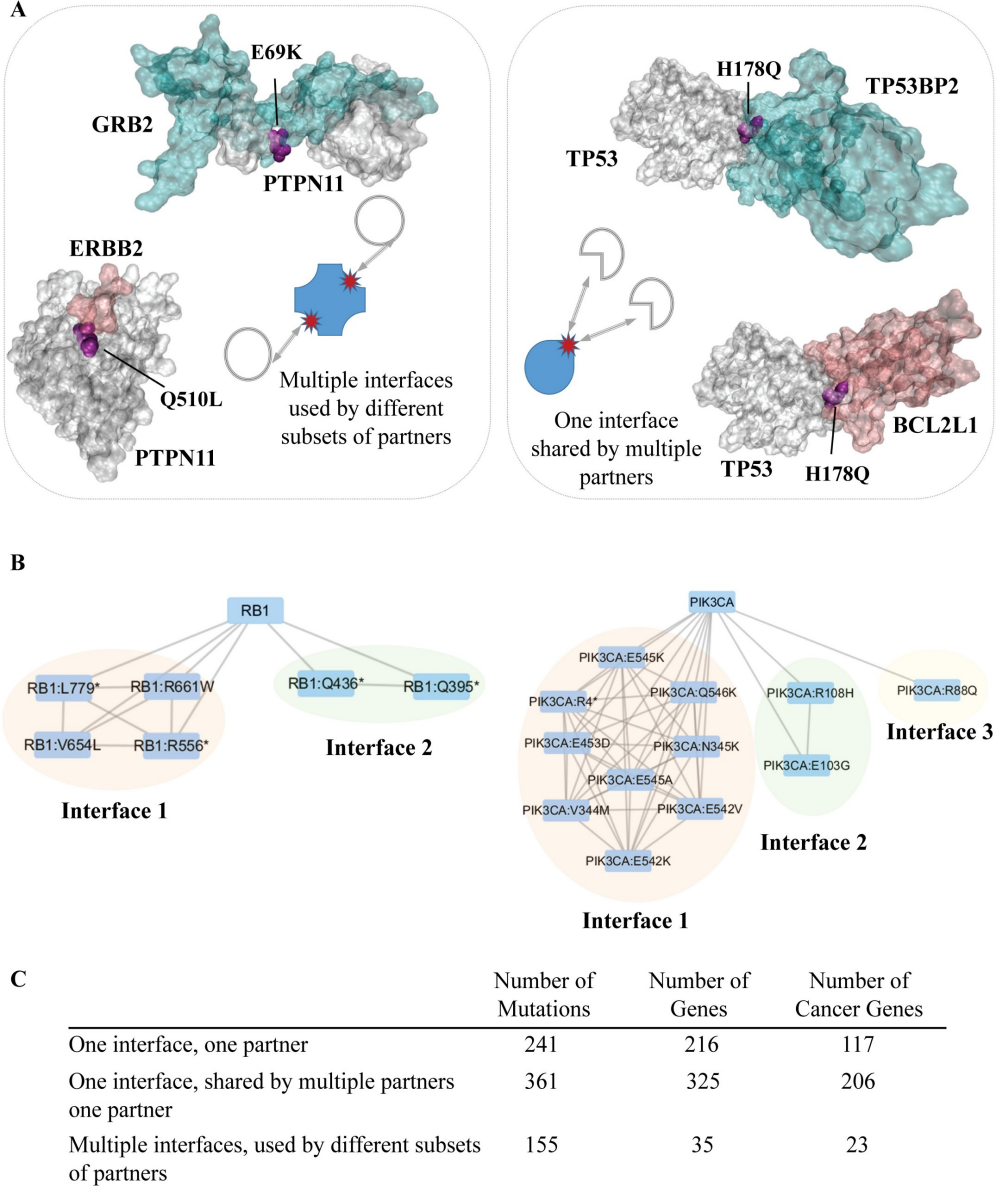
Representation of the proteins that have multiple interfaces used by different subsets of partners and one interface shared by multiple partners. (A) The left part represents an example for proteins having multiple interfaces (PTPN11) with different subset of partners and the right part represents an example for proteins having one interface (TP53) shared by different partners. (B) The organization of RB1 and PIK3CA interface mutations are represented as a network where the nodes are mutations and proteins and the edges between mutations represents at least one shared partners between two mutations. The edges between proteins and mutations represents the interface (C) The table for numbers of mutations in each interface type.

Interface mutations cover a small portion of the complete set of mutations, but these residues may affect 6144 interactions (See S1 File for detailed list for interface mutations and related interactions). This effect might result from either a single interface or multiple interfaces. We observed that some proteins have multiple interfaces, each having at least one mutation. These mutations belong to the class of “multiple interfaces used by different subsets of partners”. In Fig 5A on the left panel, we illustrated an example of this class of interfaces with their corresponding mutations. PTPN11 interacts with GRB2 and ERBB2 proteins through different interfaces. Two mutations Q510L and E69K are located on two distinct interfaces.

On the other hand, some mutations affect multiple interactions through the same interface. We call this interface class ‘one interface, shared by different partners’ and the mutations in these interfaces are ‘shared’ mutations. For instance, TP53 binds to its partner proteins TP53BP2 and BCL2L1 through overlapping regions and the mutation H178Q, located in these interfaces, is shared and may affect both interactions (see Fig 5A right panel).

Interface mutations can be better summarized with a network representation (Fig 5B). The edges between mutations represent that these mutations are located in the same or overlapping interfaces. The edges between proteins and mutations represents the interface. There are two interfaces and many mutations in the RB1 protein. There are three interfaces having multiple mutations in PIK3CA. None of the mutations in PIK3CA and RB1 is used exclusively for binding to a single partner.

The third class of interfaces are those that have only one mutation and interacts with only one partner. The table in Fig 5C represents the corresponding numbers in each class, namely ‘one interface, one partner’, ‘one interface, shared by multiple partners’ and ‘multiple interfaces, used by different subsets of partners’. A large portion of interface mutations have potential to affect multiple interactions. Interface mutations in proteins having multiple interfaces are mostly found in patches, but interface mutations in proteins with a single interface mostly remain as a singleton (P-value = 5.32x10^-43^).

**Fig 6:**
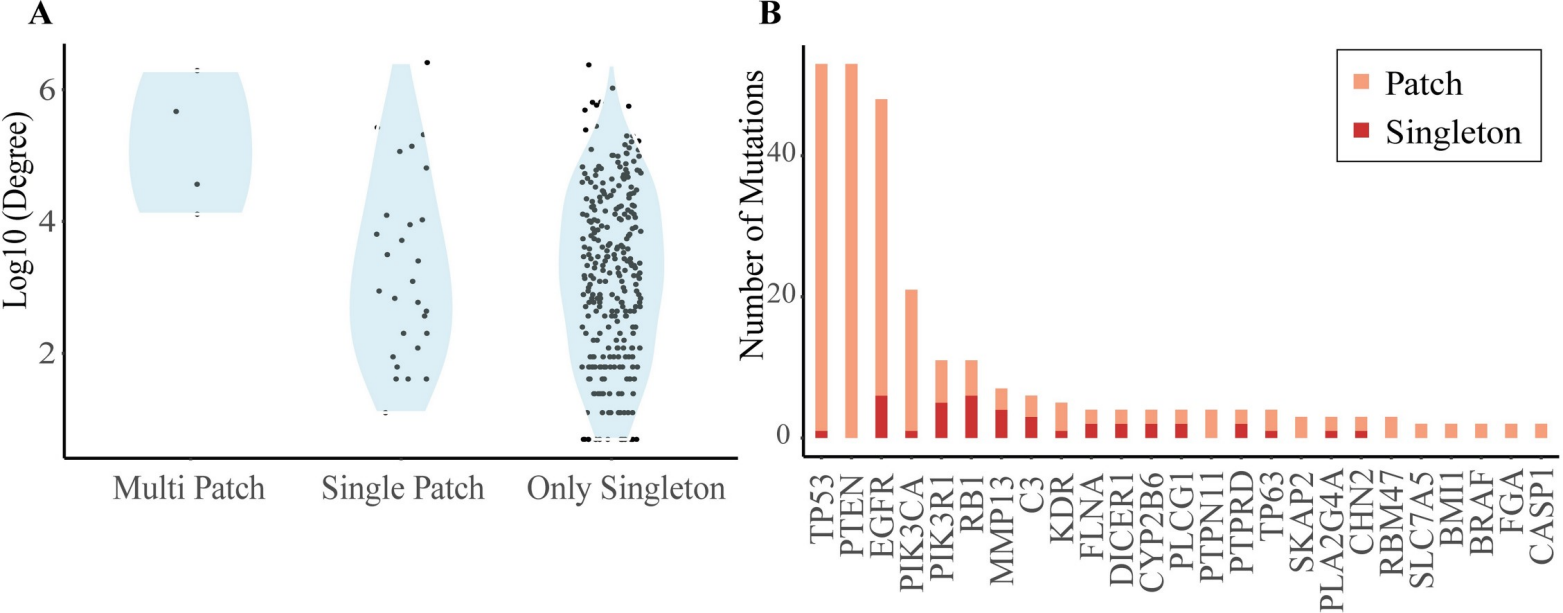
Proteins having mutations in their interfaces and the spatial arrangement of the mutations. (A) The degree distribution of the proteins in the interactome having at least one mutation in the interface region are classified based on the 3D patch organization. Three classes, namely, proteins having multiple patches, single patch and without any patch but only singleton are on the x-axis and log10 of the degree of each protein is on the y-axis. High degree proteins (hubs) in interactome have tendency to include multiple patches on their interface regions. (B) The hub proteins have more interface mutations locating inside the patches. Only the proteins having at least one patch on their interface regions are shown.

Interface mutations are likely to disrupt protein interactions that have been shown in Autism disorder [51], cancer [52]. Therefore, integrative analysis of mutations with protein networks can enhance our our knowledge of the functional effect of disease mutations [4]. When we checked the GBM mutations after mapping to the protein structures, we also found that GBM mutations are significantly more frequent in the interface region than the rest (OR = 1.1996 with P-value < 0.0001). Additionally, the number of potentially affected hub protein interactions in our analysis is 1263 through 87 highly connected proteins (on average 14.5 interactions per hub protein). The rest (4413 interactions) can be potentially affected by 3013 proteins (on average 1.47 interactions per protein). The results indicate that highly connected hub proteins tend to have multiple patches in the interface regions and interface mutations are mostly located in the patches of the hubs. Proteins with multiple patches in their interface regions are interactome hubs and are TP53, EGFR, PTEN, PIK3CA as shown in Fig 6A and 6B.

### Patient-specific sub-networks inferred from mutation profiles group tumors based on pathway similarities

As mentioned in the previous sections, mutations are rarely common in patients with GBM. Mutations may be on distinct proteins but they may alter the same pathway. Therefore, we first reconstructed patient-specific subnetworks from mutation profiles and then reduced each network into enriched pathways. We detail the outcomes of the network reconstruction and grouping patients based on network similarities in the coming section.

#### Network guided grouping of the patients and linking to clinical outcome

We used Omics Integrator software [53] to reconstruct each patients’ mutation subnetwork. Omics Integrator searches for the optimal network that connects the mutated proteins either directly or by adding intermediate nodes through high probability protein-protein interactions. The intermediate proteins are important to connect the mutated proteins and behave as a complementing component of the pathways. As a result, a sub-network was found for each patient. Reconstructed networks consist of both mutated driver genes/proteins and also intermediate proteins that link mutated proteins with high confidence edges. Networks were reconstructed and analyzed for 205 patient out of 290 patients (we lost 85 patients’ subnetworks during reconstruction and pathway enrichment steps).

A comparison of the sub-networks is essential to understand the commonalities and differences across the patients. Despite the heterogeneity, this comparison can bring out the patient groups that are similar in the network level. In general, a direct comparison of the presence of proteins and their interactions across patient subnetworks does not yield meaningful commonalities. However, many common pathways are present in these reconstructed networks and revealing these pathways is very important for a deep comparison beyond individual proteins and interactions. Therefore, we first reduced the reconstructed patient-specific networks into KEGG pathways. To focus only on pathways that are not assigned to a disease, we eliminated infections, cancers and addiction pathways. At the end, we came up with a union of 137 pathways.

**Fig 7:**
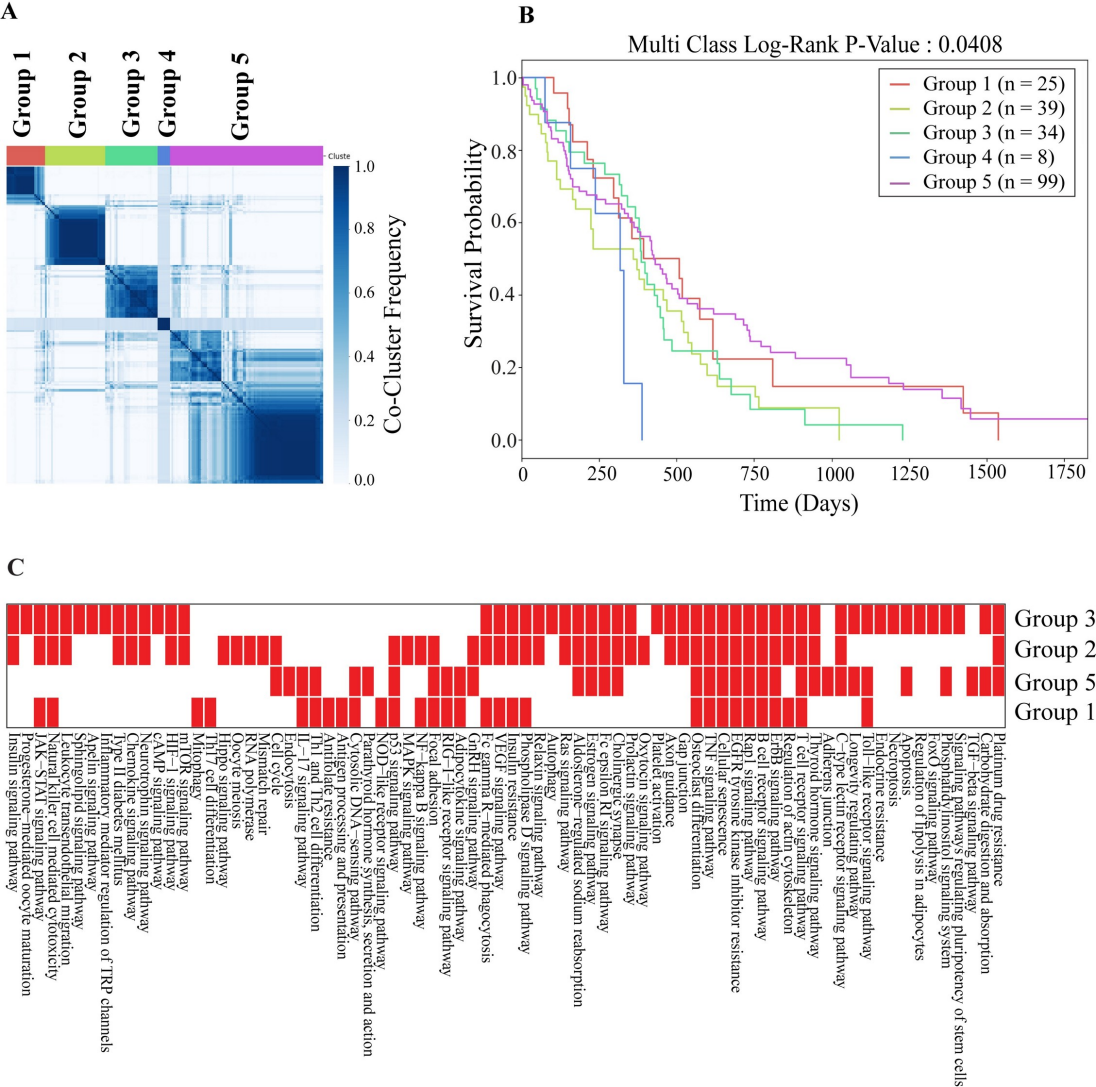
Clustering the tumors based on pathway similarities. (A) Consensus clustering of the network inferred disease signatures. Each entry in the matrix shows the co-occurence of each pair of patients. (B) Kaplan-Meier survival plots of the patient groups. Each curve represents one group. (C) Enrichment of KEGG pathways across the patient groups. Reds indicate that KEGG pathways are mainly enriched in patients of specific groups except Group 4 which does not have any KEGG pathway dominantly enriched in its patients.

We grouped patients based on similarities of the pathways inferred from networks (see Methods for the details of clustering). In Fig 7A, the consensus clustering matrix for each group is shown where the patients are consistently clustered together based on their pathway similarities. As a result, we found five groups of GBM patients containing 25, 39, 34, 8 and 99 patients, respectively.

To demonstrate the merit of network-guided analysis, we performed a similar enrichment analysis using only the mutated proteins without reconstructing patient-specific networks. We obtained significant enrichment only for 11 patients instead of 205. Only six pathways are enriched and include EGFR signaling and Glioma pathways which does not allow any further analysis to compare the patients and group them. These results also indicate that the network reconstruction from mutation profiles reveals the affected pathways more extensively.

Next, we conducted a survival analysis for each group to gain an insight into the association with the clinical outcome. Each patient’s survival status is obtained from TCGA. We searched for whether the survival curve of each group differs from each other. As shown in Fig 7B, five patient groups that are obtained by comparing enriched pathways in mutated subnetworks, significantly differ in the survival plots (log-rank test P-value = 0.0408). Among the groups, Group 4 shows the worst survival with an average of 259.75 days and Group 5 shows the best survival with an average of 450.09 days. Compared to the survival analysis obtained from the mutation profiles and mutation patches (Fig 2B), the network-guided grouping increases the coverage of the patients (162 to 206) while maintaining a substantial difference in survival across groups.

Many pathways are significantly enriched in each patient. While some pathways are significantly active in all groups of patients, many others are specific to a subset of groups. For example, mTOR signaling, Jak-Stat, and Ras signaling pathways tend to act together while the TGF-beta signaling pathway shows presence in a single group of patients. We also extracted the predominant pathways in each group of patients (Fig 7C) and found that Rap1, EGFR, and TNF signaling pathways are common in all groups. Jak-Stat pathway is present in all except Group 5. While mTOR and Hif-1 signaling pathways are present in Groups 2 and 3, TGF-beta signaling is predominant in Groups 5. Hippo signaling is only present in Group 2. We compared the three GBM subtypes (classical, preneural, mesenchymal) derived from transcriptomic data [54] with our network-based grouping. Each group is a mixture of these transcriptomic subtypes. Only Group 2 is enriched in classical subtype (P-value = 0.016/Hypergeometric Test).

Mutations in each patient are mostly located on the surface (on average 65%) and the rest is in the core and interfaces. The same trend is also observed for patient groups.

Although mTOR is mutated only in three patients in Group 5, it is present in the subnetworks of nine other patients and connects mutated proteins in the mTOR signaling pathway.

Interface mutations affect 23 interactions in Group 1, 82 interactions in Group 2, 36 interactions in Group 3, 8 interactions in Group 4 and 223 interactions in Group 5 patients. In total, interface mutations are in 318 interactions in patient networks. Out of all patients, the interactions between EGFR and MAPK8IP1, EGFR and CAV1, EGFR and RIN1 and EGFR and SHC1 proteins are the most common in 34 patients.

We illustrated a sample merged network of Group 1 in S2 Fig. The pie chart in each node represents the ratio of being mutated (red portion) or not (blue portion). The size of a node represents its frequency in Group 1. The edge thickness represents the frequency of that edge in Group 1. It is important to note that in this network there are intermediate proteins that connect mutated ones although they are not mutated. NFKBIA is an example of intermediate proteins that do not have a mutation in any patient in Group 1; however, it links many mutated proteins including IKBKB, TP53, NFKB1. Another example is CTNNB1, that is found in Group 3 as an intermediate protein to connect many mutated proteins such as PIK3R1, AKT1, LRP2. In this way, the missing parts at the pathway level can be completed and in the former example the NFKB signaling pathway, in the latter one the AKT signaling pathway can be detected in the patient groups.

There are in total of 971 proteins in the union network of patient groups. The majority of proteins are unique to patient groups. Very few (17 proteins) are common in all groups, whereas many of them (685 proteins) are present in only one patient group (Group 4 was excluded because it was very small). One of the common proteins is TP53 which is also a hub protein (node centrality). We ranked the proteins in each merged network of patient groups based on the node centrality. However, some central proteins are specific to a patient group; such as IKBKG in Group 1, or MDM2, which is a central protein only in Group 2.

### Connecting the patient groups to drug sensitivity

The significant association between patient groups and survival led us to further analyze the possible therapeutic targets in each group. The therapeutic information is very sparse in TCGA; therefore, we collected drug sensitivity data of the GBM cell lines treated with different drugs from CancerRxGene [55]. We also retrieved the target proteins and the target pathways of each drug. As a result, we collected 37 GBM Cell Lines having the mutation profile information in the Cell Model Passports database [56]. In total, there were 13243 mutations. We got the intersection of these mutations with the set of GBM mutations we used in our study and found that 23 mutations are common of which 16 are located in patches. These enriched patches are on PTEN, TP53, EGFR, BRAF and RB1 proteins. As a result, we obtained 17 cell lines treated with 73 drugs that target 18 pathways.

To link the patient groups to the drug response data of each cell line, we used the signature 3D patches. We found that 44 patches tend to be significantly present in one or multiple patient groups including PTEN, TP53, EGFR, BRAF and RB1 patches which are also present in cell lines (S3 Fig). According to our results, all patches of PIK3R1, PTEN, TP53 and one patch of PIK3CA and BRAF have a strong tendency to be present in Group 5. While RB1, TP53, PTEN, patches are enriched in Group 2; TP53, PTEN patches without EGFR and RB1 have a bias to be in Group 1. EGFR has 3 patches, and PTEN has two patches which are present in distinct groups. While all patches of EGFR are found only in Group 5, Groups 4 and 3 only have one of the patches of EGFR. Patch 1, 3 and 4 of PIK3CA are only found in Group 3, Patch 2 of PIK3CA is found in Groups 2, 3 and 5. One of the very well characterized biomarkers in GBM is BRAF. BRAF mutations V600E and G596D form a 3D patch in only Group 5.

**Fig 8:**
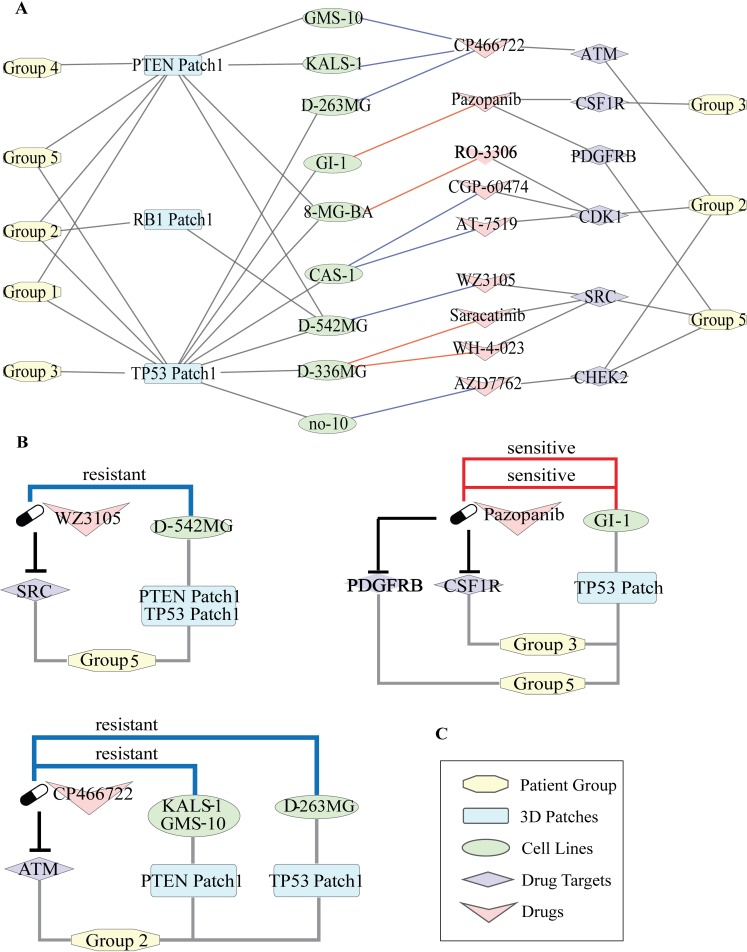
Linking the network-guided patient groups to drug treatments through 3D spatial patches. (A) Nodes represent group identifiers, patch names, cell line names, drugs and their targets, respectively. Edges between group Ids and patch names imply the presence of the presence of the corresponding patch in the group. Edges between patch names and cell line Ids represent that at least one mutation in the corresponding patch is present in the connected cell line. Edges between cell lines and drug names imply that the cell line is treated with the corresponding drug. If the cell line is sensitive to the drug then the edge color is red, if resistant the edge color is blue. Edges between drugs and target proteins are to show that proteins targeted by the corresponding drugs that are significantly present in the linked group. (B,C,D) Three submodules are retrieved from the network to show some therapeutic hypotheses where the first one is Pazopanib (targeting CSF1R and PDGFRB) for Group 5, the second is the possible resistance of Group 2 to ATM inhibition and the last one is WZ3105 (targeting SRC) for Group 5.

Group1 is linked to the cell lines having at least one mutation in TP53 Patch and PTEN Patch1, Group 2 is linked to the cell lines having TP53 Patch and PTEN Patch1 and also RB1 Patch. Group 4 has the PTEN Patch and Group 5 has EGFR Patch2, TP53 Patch, PTEN Patch1 together with the BRAF Patch (Fig 8A).

Our first therapeutic hypothesis is based on Pazopanib which is a multi-targeted receptor tyrosine kinase inhibitor. Pazopanib is linked to our patient groups through its targets CSF1R and PDGFRB that are significantly enriched in Group 3 and Group 5, respectively (Fig 8B). GBM cell line GI-1 is sensitive to Pazopanib. GI-1 has at least one mutation in TP53 patch which is predominantly available in Group 3 and Group 5. CSF1 (colony stimulating factor 1) binds to CSF1R and activates several signaling pathways, including Ras/Raf/MAPK, phosphatidylinositol 3-kinase (PI3-kinase) and JAK/STAT pathways. When we refer to the pathway enrichment results in Group 3, Phosphatidylinositol and JAK-STAT pathways were enriched. Additionally, CSF1R is also on tumor-associated macrophages and microglia (TAMs) which are highly available in glioma microenvironment. CSF1, the ligand of CSF1R, is responsible for the differentiation of TAMs to pro-tumorigenic. The inhibition of CSF1R results in the differentiation of the macrophages and makes them more anti-tumorigenic. Another target of Pazopanib is PDGFRB protein is a receptor tyrosine kinase and functions as a cell surface receptor. It activates cell proliferation and survival. Moreover, it is proven that PDGFRB is overexpressed in GBM cells and very important for self-renewal [57]. Therefore, we suggest that Group 3 and Group 5 might be sensitive to a treatment based on Pazopanib.

Another example of a significant target is ATM, which is a target of ATM-inhibitor (CP466722). ATM is present in the network of Group 2 where PTEN Patch 1 is enriched (Fig 8C). Two GBM cell lines (KALS-1, GMS-10) are resistant to this drug molecule. ATM is a mediator of PTEN phosphorylation and ATM targeting drugs are used to make a patient more sensitive to radiotherapy. In our therapeutic hypotheses, we suggest that Group 2 might be resistant to ATM-dependent therapy.

Our last example is SRC protein as a target in Group 5 which is a non-receptor protein tyrosine kinase and plays an important role in many cellular processes such as growth, adhesion, and differentiation. It is also a component of several cell signaling pathways including EGFR, ERBB, and Rap1 signaling pathways. Group 5 is associated with the GBM cell line D-452MG through TP53 and PTEN patches. The kinase-inhibitor WZ3105, that targets SRC, is resistant in D-452MG and we suggest that Group 5 might be possibly resistant to WZ3105 according to our therapeutic hypothesis (Fig 8D).

## Discussion

The mutation landscape of GBM tumors is very heterogeneous and not discriminative to classify disease progression and subtypes. Given the impact of mutations in protein interactions and eventually cellular signaling pathways, reverse engineering from mutation profiles to patient-specific subnetworks can shed light on network-level changes to observe hidden commonalities. Therefore, we applied a systems-level strategy to the patient-derived information of 290 GBM tumors to gather knowledge about their commonalities. We first started with an in-depth analysis of individual mutations such as their spatial organization, physicochemical characteristics, and their effects in binding. Our results show that out of 15399 mutations, 4702 mutations have structural information and 10% of these mutations are spatially grouped into patches, while most mutations are spatially distant to other mutations, namely, singletons. Interestingly, distinct patches of a protein are located in distinct domains that could have distinct functional consequences. Despite a small portion of all mutations, 3D patches reduce the heterogeneity across patients and more commonalities can be identified. To show the success of the patches in overcoming heterogeneity, we associated them with the survival data. Indeed, grouping patients based on the patch information significantly discriminates the survival curves. For example, tumors in patients with at least one mutation in PI3K patches are more aggressive compared to the tumors with at least one mutation in TP53 patch. These results are a proof of concept that 3D spatial grouping of mutations can be related to clinical outcome and is useful in overcoming heterogeneity.

In our follow-up analyses, we found that GBM mutations are significantly more frequent in interface regions than the rest. Although a small portion of all mutations is located in the core region; they may affect the function more severely. Mutations in the core tend to preserve their chemical classes while interface and surface mutations are significantly more prone to changes. Mutated residues are mostly populated on the surface and their functional effects are less severe than the rest (interface and core). When we limit our analysis to only the mutations on cancer-driver genes, we still have the same results. Furthermore, tumor suppressors have large patches while oncogenes have multiple and relatively smaller patches. Having the mutations on very specific sites of oncogenes agrees with the known observation that making a protein more active is harder. On the other hand, tumor suppressors can be functionally damaged in several ways thus their mutations could be distributed in large regions. Also, nonsense and frameshift mutations are more frequent in tumor suppressors.

Mutations in hub proteins are organized into very large patches that connect mutations in multiple binding sites through the core of the protein (observed in tumor suppressors). Additionally, the organization of multiple 3D patches in hub proteins implies the importance of 3D patches in cancer vulnerability (observed in oncogenes). 3D spatial organization of mutations in hub proteins may provide a fitness advantage to tumor cells. Not all mutations are equally damaging. Some mutations are neutral and some mutations cause damage in protein stability or protein binding. Our results suggest that hub proteins’ patch mutations are more disease-causing, whereas other proteins’ singleton mutations are more disease-causing.

Some proteins repeatedly use a single interface to interact with their partners while some proteins have multiple interfaces. The characteristics of the mutations are also different in these interfaces. Interface mutations in proteins with a single interface stay distant from other mutations and are usually present as singletons; however, interface mutations in proteins having multiple interfaces are mostly located in patches.

When we analyzed the patient-specific networks and the consensus network of these patients, we observed that although mutation profiles are very heterogeneous across patients and their pathway-level representation is very limited, the network-based analysis groups the patients better and reveals predominant pathways in each group. Additionally, the network-based similarity analysis shows that each group of patients carries a set of signature 3D mutation patches. For example, EGFR, TP53, PIK3CA, PIK3R1 patches are frequently found in Group 3, TP53, RB1, PTEN patches in Group 2. Beyond the list of mutations, the network-guided analysis also reveals similarities across patients and overcomes the heterogeneity in mutation profiles by completing the interaction components that mutated proteins potentially affect. We found that there are significant differences across the patient groups in their survival. Additionally, several pathways are common in each patient group, such as the Jak-Stat pathway being enriched in three groups, the TGF-beta signaling pathway being present in only one patient group. This pathway-level outcome led us to link the available drug treatment data to our patient groups. Because the drug treatment data is very sparse in TCGA, we used the GBM cell lines for this purpose. Each group of patients is linked to each GBM cell line through its predominant patches. For example, we found that PDGFRB, the target of Pazopanib, is significantly present in the subnetworks of Group 5 which has the TP53 patch as the marker. GBM cell lines having a mutation in the TP53 patch and treated with Pazopanib are sensitive to this drug. We therefore proposed that Pazopanib may be efficient in Group 5. This sort of therapeutic hypotheses was found and suggested for each group of patients as provided in Figs 8 and S4.

Overall, these results show that network-guided interpretation of mutations and their 3D organizations give a deeper insight into their impact and useful in overcoming the inter-tumor heterogeneity that is the main barrier in finding optimal treatment strategies. Despite the apparent diversity in the mutation profiles of GBM tumors, the 3D spatial grouping of mutations and network-guided clustering of tumors reveal several commonalities and enable us to link the gathered information to clinical outcomes and therapeutic data. Our approach from mutations to protein interactions and eventually to signaling networks and pathways transforms the tumor information into clinically interpretable knowledge. We believe that this study represents a good example of how networks can be used efficiently in precision medicine.

## Methods

### Data collection and preprocessing

The missense, nonsense and frameshift mutations in Glioblastoma were retrieved from TCGA which has been published in [58] for 290 patients. First, all proteins that have at least one mutation was searched in PDB [33]. If a structure was not available, we made use of ModBase [34] homology models. The structural information of the protein interactions was collected from PDB, Interactome3D [7], PRISM [44] and Interactome Insider [12]. PDB deposits protein complexes that are crystallized together. Interactome3D predicts the protein complexes through structure and domain similarity with a template structure. PRISM uses known interfaces to predict new protein interactions. We used the pre-runned PRISM results for a subset of the proteome, rather than the whole proteome. The other source for structural protein interactions was the Interactome Insider. It produces the binding sites on each partner of the protein interaction. Different than PRISM and Interactome3D, it does not give the structure or the pose of the predicted protein complex. We also downloaded the human proteome from UniProt [59] for cross-referencing from one data source to another. Additionally, the residue positions in sequence are not consistent with the residue positions in protein structures. A PDB entry or a homology model of a UniProt sequence may represent only a fragment of the given protein and the residue numbering may not be the same with the sequence positions. Therefore, we performed UniProt sequence to the sequence in the protein structure alignment to find the exact position of each residue.

Moreover, we retrieved the known cancer genes from The Network of Cancer Genes [38], cancer-related genes from Cancer Gene Census of The Catalogue of Somatic Mutations in Cancer (COSMIC) [40], validated oncogenic mutations from Cancer Genome Interpreter [39]. The Network of Cancer Genes is a repository for predicted or known cancer driver genes that have been manually curated. In this analysis, we only used the known cancer genes. Similarly, Cancer Gene Census of COSMIC includes the manually curated cancer genes that behave as driver effect for human cancer. We included these genes into our analysis. On the other hand, dataset of Cancer Genome Interpreter gives the oncogenic mutations by using the information from DoCM, ClinVar, OncoKB, and IARC. We also took these cancer driver mutations. Additionally we obtained the mutation information for GBM from Broad Institute FireBrowser which includes MutSig2CV v3.1 [42], Mutation Assessor [43], CHASM 1.0.5 [41]. Mutation Assessor only considers the missense mutations and gives the gene names which missense mutations on and their functional impacts. In our analysis, we only considered the high and medium functional impact genes as significant genes. Secondly, MutSig2CV gives the gene significance according to mutations on the gene. In this project, we took the genes whose P-value is smaller than 0.05 as significant genes. Lastly, CHASM uses the missense mutations and gives the probability for each mutation due to the selective survival advantage that is provided to the cancer cells by the mutation. In this analysis, we only considered the mutations that have P-value smaller than 0.05. To reach the gene name for the mutation in CHASM, we also needed Ensembl BioMart [60] for the conversion between RefSeq mRNA ID to Ensembl Transcript ID to reach the official gene symbol. Finally, the confidence weighted interactome deposited in iRefWeb has been downloaded for the reconstruction of patient-specific sub-networks inferred from mutation profiles of each patient. Afterward, the interactions having structural information also retrieved for constructing structural interactome for Omics Integrator analysis.

### Identification of the spatial clusters and grouping the patients

Each cancer-related driver protein structure and protein complexes were converted into a network of residue-residue interactions. If any atom in a residue is in close proximity to any atom in another residue, then these two residues were considered to interact. The proximity was defined as the distance of less than 5Å between any atoms. We constructed a residue contact graph R(v, e) for each structure where v is the set of residues and e is the set of edges between these residues. We searched for all shortest paths between each mutated residue pairs with a length of less than 3 to identify the spatial clusters, which means that if two mutated residues are either directly connected or only one residue is in between them. Then all the extracted shortest paths merged to create a subgraph P(v’,e’) representing one spatial cluster, namely “patch” where v’⊂v and e’⊂e. Mutations that are not assigned to a patch were labelled as singletons, meaning that these residues are distant to other mutations in the same protein.

Patients are grouped based on the presence of each patch. This grouping is performed iteratively. The first group is formed by the patient having at least one mutation in the most frequent patch. The second group is the patients having at least one mutation in the second most frequent patch, and having not any mutation in the most frequent patch. This iteration continues until each group has at least ten patients. In this way, each group is mutually exclusive, where there are no common patients across the groups.

### Identification of protein regions and the effect of the mutations

Proteins can be divided into three regions, namely, the core, surface and interface regions. The conventional approach for identifying these regions is to calculate solvent-accessible surface areas of each residue in the protein. FreeSASA [61] is a software designed for calculation of solvent accessible surface area at both residue level and molecule level. In general, if the relative solvent accessible surface area of a residue in its monomer state is greater than or equal to 5%, then this residue is labelled as the surface residue. Interface residues that are collected from structural interactome are excluded from the surface residue set. The rest is identified as core residues. However, we only considered the structure files whose length greater than 50 residues for this analysis.

We used EVmutation and PolyPhen-2 web servers to calculate the effect of mutations if they are damaging or neutral. The EVmutation data provided the information for a limited number of proteins in text format where each position in a UniProt entry is substituted by the remaining 19 amino acid and the damage score is calculated. The more negative values of the calculated score means the more damaging mutation. The details of the calculation steps of EVmutation scores are in reference [49]. On the other hand, Polyphen2 gives the results as being probably damaging, possibly damaging or benign. We used mutation effect data to compare the damage of the mutations based on their localization and their role in the cancer progression (tumor suppressors or oncogenes).

### Sub-network reconstruction for each patient

In our setup, we focused on driver genes to reduce the noise caused by passenger mutations. We added each protein having at least one nonsynonymous mutation (missense, nonsense and frameshift) on a driver gene/protein in a tumor sample to the list as the base for network reconstruction and weighted each protein with their number of mutations. We used the probability-weighted protein-protein interactions in iRefWeb [62] as the reference interactome. This reference interactome further is filtered by the interactions having structural information. Additionally, Omics Integrator has a unique feature to avoid biasing the dominance of well-studied proteins or hubs in the final network. We used hub-penalizing parameters set to reveal more specific pathways for a better comparison of the patient-specific networks. Two different values of the scaling factor of hub proteins (μ parameter, described in Methods) were used for this purpose and resulting optimal networks were merged. Omics Integrator software was used to reconstruct patient-specific sub-networks. Given a reference graph G(V, E, w) where V is the node set {v|v ∈V}, E is the edge set {e|e ∈ E} and w is the edge weights, the Forest module of Omics Integrator solves the prize-collecting Steiner forest problem for a given set of nodes with predefined prizes. In our case, the terminal nodes were the mutated cancer proteins for each patient and the prizes were given according to the significance of the mutations included in each protein. If the mutation on the terminal node was significant, we added 1 as a prize to the terminal node and if it was not significant, the added prize was 0.5. Therefore, the prize list composed of the proteins from cancer genes having at least one mutation. We retrieved the iRefWeb v8.0 interactome and filtered the interactions if they have structural information or not. Therefore, we used structural iRefWeb interactome as the weighted reference interactome in our modeling. To have a stringent setup, we filtered out interactions having a score less than 0.4 and also the proteins such as UBC, APP, ELAVL1, SUMO2, CUL3 and the proteins huge in size (TTN, MUC16, SYNE1, NEB, MUC19, CCDC168, FSIP2, OBSCN, GPR98) to limit the noise coming from random mutations in these proteins as in [63]. The parameter set ω (omega) = 10.0, depth (D) = 6 and β (beta) = 10 was used for the reconstruction. Omega (ω) parameter was used for tuning the number of trees in the final network, depth is the number of edges from the root to the leaf nodes and beta (β) is a scaling factor to force more prize nodes to enter the final network. Finally, mu (μ) is another scaling factor to tune the dominance of hub proteins in the final network. We used two mu (μ) values (0.005. 0.01) to recover the canonical pathways and more specific ones and merged the node and edge set of the reconstructed networks to come up with a single network for each patient.

### Network-guided grouping of the patients

WebgestaltR [64] package evaluated each patient’s subnetwork to obtain the overrepresented KEGG pathways in each network. Pathways were assumed to be enriched in the sub-network if the False Discovery Rate (FDR) is less than 0.1. In the resulting list of pathways, we eliminated disease pathways including infections, cancer, addiction related pathways. Then we prepared a matrix where rows are union set of enriched pathways, columns are patient barcodes and entries are the enrichment score (ES) of a pathway in the corresponding barcode’s sub-network. If the pathway is not enriched, 0 is inserted into that entry. We used this matrix for implementing the non negative matrix factorization without a network regularizer and then consensus clustering from pyNBS package which is a Python implementation of NBS [65]. The identified groups were searched for if any identified spatial patch tends to represent a group using hypergeometric testing.

### Linking the patient groups to drug response

We linked each patient group to the GBM cell lines retrieved from Cell Model Passports through the mutation information. If at least one mutation belonging to a predominant patch in a group is also present in the GBM cell line then the patient group is connected with that cell line. The drug treatment data is obtained from CancerRxGene [55] where the sensitivity of the drugs to the cell lines are deposited. For each drug, target proteins and target pathways are also obtained.

## Supporting information

S1 File Interface mutations and the interactions that are related for each mutation. For each 757 interface mutations, corresponding interactions that are related by the mutation are listed in tab separated format.
(TXT)

S1 Fig (A) Grouping patients based on individual mutations. Each column represents a patient and each row represents a mutation. (B) Association between Kaplan-Meier survival curves and patient groups by the most frequent individual mutations.
(TIF)

S2 Fig The merged network of Group 1. The nodes are labelled with a pie chart colored in red and/or blue color. The fraction of the red color represents the count of being a mutated protein in the patient network. The fraction of the blue color represents the count of being an intermediate protein connecting mutated ones in the patient network. Cytoscape is used for network visualization.
(TIF)

S3 Fig Predominant 3D patches in each patient group. Columns are patches and rows are patient groups. Red color represents the presence of the corresponding patch in the patient group.
(TIF)

S4 Fig (A, B) Additional therapeutic hypotheses where the first one is RO-3306 (targeting CDK1) for Group 2, the second is the possible resistance of Group 2 and Group 5 to CHEK2 inhibition by AZD7762.
(TIF)

## References

[1] Tomczak K, Czerwinska P, Wiznerowicz M. The Cancer Genome Atlas (TCGA): an immeasurable source of knowledge. Contemp Oncol (Pozn). 2015;19(1A):A68–77. Epub 2015/02/19. 10.5114/wo.2014.47136 25691825 PMC4322527

[2] Zhang J, Baran J, Cros A, Guberman JM, Haider S, Hsu J, et al International Cancer Genome Consortium Data Portal—a one-stop shop for cancer genomics data. Database (Oxford). 2011;2011:bar026 Epub 2011/09/21. 10.1093/database/bar026 21930502 PMC3263593

[3] Nussinov R, Jang H, Tsai CJ, Cheng F. Review: Precision medicine and driver mutations: Computational methods, functional assays and conformational principles for interpreting cancer drivers. PLoS Comput Biol. 2019;15(3):e1006658 Epub 2019/03/29. 10.1371/journal.pcbi.1006658 30921324 PMC6438456

[4] Sahni N, Yi S, Taipale M, Fuxman Bass JI, Coulombe-Huntington J, Yang F, et al Widespread macromolecular interaction perturbations in human genetic disorders. Cell. 2015;161(3):647–60. Epub 2015/04/25. 10.1016/j.cell.2015.04.013 25910212 PMC4441215

[5] Buljan M, Blattmann P, Aebersold R, Boutros M. Systematic characterization of pan-cancer mutation clusters. Mol Syst Biol. 2018;14(3):e7974 Epub 2018/03/25. 10.15252/msb.20177974 29572294 PMC5866917

[6] Kar G, Gursoy A, Keskin O. Human cancer protein-protein interaction network: a structural perspective. PLoS Comput Biol. 2009;5(12):e1000601 Epub 2009/12/17. 10.1371/journal.pcbi.1000601 20011507 PMC2785480

[7] Mosca R, Ceol A, Aloy P. Interactome3D: adding structural details to protein networks. Nat Methods. 2013;10(1):47–53. Epub 2013/02/13. 10.1038/nmeth.2289 . 23399932

[8] Porta-Pardo E, Garcia-Alonso L, Hrabe T, Dopazo J, Godzik A. A Pan-Cancer Catalogue of Cancer Driver Protein Interaction Interfaces. PLoS Comput Biol. 2015;11(10):e1004518 Epub 2015/10/21. 10.1371/journal.pcbi.1004518 26485003 PMC4616621

[9] Orchard S, Kerrien S, Abbani S, Aranda B, Bhate J, Bidwell S, et al Protein interaction data curation: the International Molecular Exchange (IMEx) consortium. Nat Methods. 2012;9(4):345–50. Epub 2012/03/29. 10.1038/nmeth.1931 22453911 PMC3703241

[10] Del-Toro N, Duesbury M, Koch M, Perfetto L, Shrivastava A, Ochoa D, et al Capturing variation impact on molecular interactions in the IMEx Consortium mutations data set. Nat Commun. 2019;10(1):10 Epub 2019/01/04. 10.1038/s41467-018-07709-6 . 30602777 PMC6315030

[11] Gao J, Chang MT, Johnsen HC, Gao SP, Sylvester BE, Sumer SO, et al 3D clusters of somatic mutations in cancer reveal numerous rare mutations as functional targets. Genome Med. 2017;9(1):4 Epub 2017/01/25. 10.1186/s13073-016-0393-x 28115009 PMC5260099

[12] Meyer MJ, Beltran JF, Liang S, Fragoza R, Rumack A, Liang J, et al Interactome INSIDER: a structural interactome browser for genomic studies. Nat Methods. 2018;15(2):107–14. Epub 2018/01/23. 10.1038/nmeth.4540 29355848 PMC6026581

[13] Nishi H, Tyagi M, Teng S, Shoemaker BA, Hashimoto K, Alexov E, et al Cancer missense mutations alter binding properties of proteins and their interaction networks. PLoS One. 2013;8(6):e66273 Epub 2013/06/27. 10.1371/journal.pone.0066273 23799087 PMC3682950

[14] Nussinov R, Jang H, Tsai CJ, Cheng F. Precision medicine review: rare driver mutations and their biophysical classification. Biophys Rev. 2019 Epub 2019/01/06. 10.1007/s12551-018-0496-2 . 30610579 PMC6381362

[15] An O, Gursoy A, Gurgey A, Keskin O. Structural and functional analysis of perforin mutations in association with clinical data of familial hemophagocytic lymphohistiocytosis type 2 (FHL2) patients. Protein Sci. 2013;22(6):823–39. Epub 2013/04/18. 10.1002/pro.2265 23592409 PMC3690721

[16] Engin HB, Kreisberg JF, Carter H. Structure-Based Analysis Reveals Cancer Missense Mutations Target Protein Interaction Interfaces. PLoS One. 2016;11(4):e0152929 Epub 2016/04/05. 10.1371/journal.pone.0152929 27043210 PMC4820104

[17] Ozdemir ES, Gursoy A, Keskin O. Analysis of single amino acid variations in singlet hot spots of protein-protein interfaces. Bioinformatics. 2018;34(17):i795–i801. Epub 2018/11/14. 10.1093/bioinformatics/bty569 . 30423104

[18] Ozdemir ES, Halakou F, Nussinov R, Gursoy A, Keskin O. Methods for Discovering and Targeting Druggable Protein-Protein Interfaces and Their Application to Repurposing. Methods Mol Biol. 2019;1903:1–21. Epub 2018/12/14. 10.1007/978-1-4939-8955-3_1 . 30547433 PMC8185533

[19] Zhao J, Cheng F, Wang Y, Arteaga CL, Zhao Z. Systematic Prioritization of Druggable Mutations in approximately 5000 Genomes Across 16 Cancer Types Using a Structural Genomics-based Approach. Mol Cell Proteomics. 2016;15(2):642–56. Epub 2015/12/15. 10.1074/mcp.M115.053199 26657081 PMC4739678

[20] Acuner Ozbabacan SE, Gursoy A, Nussinov R, Keskin O. The structural pathway of interleukin 1 (IL-1) initiated signaling reveals mechanisms of oncogenic mutations and SNPs in inflammation and cancer. PLoS Comput Biol. 2014;10(2):e1003470 Epub 2014/02/20. 10.1371/journal.pcbi.1003470 24550720 PMC3923659

[21] Engin HB, Guney E, Keskin O, Oliva B, Gursoy A. Integrating structure to protein-protein interaction networks that drive metastasis to brain and lung in breast cancer. PLoS One. 2013;8(11):e81035 Epub 2013/11/28. 10.1371/journal.pone.0081035 24278371 PMC3838352

[22] Tuncbag N, Kar G, Gursoy A, Keskin O, Nussinov R. Towards inferring time dimensionality in protein-protein interaction networks by integrating structures: the p53 example. Mol Biosyst. 2009;5(12):1770–8. Epub 2009/07/09. 10.1039/B905661K 19585003 PMC2898629

[23] Kamburov A, Lawrence MS, Polak P, Leshchiner I, Lage K, Golub TR, et al Comprehensive assessment of cancer missense mutation clustering in protein structures. Proc Natl Acad Sci U S A. 2015;112(40):E5486–95. Epub 2015/09/24. 10.1073/pnas.1516373112 26392535 PMC4603469

[24] Niu B, Scott AD, Sengupta S, Bailey MH, Batra P, Ning J, et al Protein-structure-guided discovery of functional mutations across 19 cancer types. Nat Genet. 2016;48(8):827–37. Epub 2016/06/14. 10.1038/ng.3586 27294619 PMC5315576

[25] Drake JM, Paull EO, Graham NA, Lee JK, Smith BA, Titz B, et al Phosphoproteome Integration Reveals Patient-Specific Networks in Prostate Cancer. Cell. 2016;166(4):1041–54. Epub 2016/08/09. 10.1016/j.cell.2016.07.007 27499020 PMC4985183

[26] Kim YA, Wuchty S, Przytycka TM. Identifying causal genes and dysregulated pathways in complex diseases. PLoS Comput Biol. 2011;7(3):e1001095 Epub 2011/03/11. 10.1371/journal.pcbi.1001095 21390271 PMC3048384

[27] Tuncbag N, Milani P, Pokorny JL, Johnson H, Sio TT, Dalin S, et al Network Modeling Identifies Patient-specific Pathways in Glioblastoma. Sci Rep. 2016;6:28668 Epub 2016/06/30. 10.1038/srep28668 27354287 PMC4926112

[28] Hofree M, Shen JP, Carter H, Gross A, Ideker T. Network-based stratification of tumor mutations. Nat Methods. 2013;10(11):1108–15. Epub 2013/09/17. 10.1038/nmeth.2651 24037242 PMC3866081

[29] Zhong X, Yang H, Zhao S, Shyr Y, Li B. Network-based stratification analysis of 13 major cancer types using mutations in panels of cancer genes. BMC Genomics. 2015;16 Suppl 7:S7 Epub 2015/06/24. 10.1186/1471-2164-16-S7-S7 26099277 PMC4474538

[30] Engin HB, Hofree M, Carter H. Identifying mutation specific cancer pathways using a structurally resolved protein interaction network. Pac Symp Biocomput. 2015:84–95. Epub 2015/01/17. 25592571 PMC4299875

[31] Cerami E, Demir E, Schultz N, Taylor BS, Sander C. Automated network analysis identifies core pathways in glioblastoma. PLoS One. 2010;5(2):e8918 Epub 2010/02/20. 10.1371/journal.pone.0008918 20169195 PMC2820542

[32] Sidiropoulos K, Viteri G, Sevilla C, Jupe S, Webber M, Orlic-Milacic M, et al Reactome enhanced pathway visualization. Bioinformatics. 2017;33(21):3461–7. Epub 2017/10/28. 10.1093/bioinformatics/btx441 29077811 PMC5860170

[33] Rose PW, Prlic A, Altunkaya A, Bi C, Bradley AR, Christie CH, et al The RCSB protein data bank: integrative view of protein, gene and 3D structural information. Nucleic Acids Res. 2017;45(D1):D271–D81. Epub 2016/10/30. 10.1093/nar/gkw1000 27794042 PMC5210513

[34] Pieper U, Eswar N, Braberg H, Madhusudhan MS, Davis FP, Stuart AC, et al MODBASE, a database of annotated comparative protein structure models, and associated resources. Nucleic Acids Res. 2004;32(Database issue):D217–22. Epub 2003/12/19. 10.1093/nar/gkh095 14681398 PMC308829

[35] Leiserson MD, Vandin F, Wu HT, Dobson JR, Eldridge JV, Thomas JL, et al Pan-cancer network analysis identifies combinations of rare somatic mutations across pathways and protein complexes. Nat Genet. 2015;47(2):106–14. Epub 2014/12/17. 10.1038/ng.3168 25501392 PMC4444046

[36] Chalhoub N, Baker SJ. PTEN and the PI3-kinase pathway in cancer. Annu Rev Pathol. 2009;4:127–50. Epub 2008/09/05. 10.1146/annurev.pathol.4.110807.092311 18767981 PMC2710138

[37] Georgescu MM. PTEN Tumor Suppressor Network in PI3K-Akt Pathway Control. Genes Cancer. 2010;1(12):1170–7. Epub 2011/07/23. 10.1177/1947601911407325 21779440 PMC3092286

[38] Repana D, Nulsen J, Dressler L, Bortolomeazzi M, Venkata SK, Tourna A, et al The Network of Cancer Genes (NCG): a comprehensive catalogue of known and candidate cancer genes from cancer sequencing screens. Genome Biol. 2019;20(1):1 Epub 2019/01/05. 10.1186/s13059-018-1612-0 30606230 PMC6317252

[39] Tamborero D, Rubio-Perez C, Deu-Pons J, Schroeder MP, Vivancos A, Rovira A, et al Cancer Genome Interpreter annotates the biological and clinical relevance of tumor alterations. Genome Med. 2018;10(1):25 Epub 2018/03/30. 10.1186/s13073-018-0531-8 29592813 PMC5875005

[40] Sondka Z, Bamford S, Cole CG, Ward SA, Dunham I, Forbes SA. The COSMIC Cancer Gene Census: describing genetic dysfunction across all human cancers. Nat Rev Cancer. 2018;18(11):696–705. Epub 2018/10/08. 10.1038/s41568-018-0060-1 30293088 PMC6450507

[41] Carter H, Samayoa J, Hruban RH, Karchin R. Prioritization of driver mutations in pancreatic cancer using cancer-specific high-throughput annotation of somatic mutations (CHASM). Cancer Biol Ther. 2010;10(6):582–7. Epub 2010/06/29. 10.4161/cbt.10.6.12537 20581473 PMC3040948

[42] Lawrence MS, Stojanov P, Polak P, Kryukov GV, Cibulskis K, Sivachenko A, et al Mutational heterogeneity in cancer and the search for new cancer-associated genes. Nature. 2013;499(7457):214–8. Epub 2013/06/19. 10.1038/nature12213 23770567 PMC3919509

[43] Reva B, Antipin Y, Sander C. Predicting the functional impact of protein mutations: application to cancer genomics. Nucleic Acids Res. 2011;39(17):e118 Epub 2011/07/06. 10.1093/nar/gkr407 21727090 PMC3177186

[44] Tuncbag N, Gursoy A, Nussinov R, Keskin O. Predicting protein-protein interactions on a proteome scale by matching evolutionary and structural similarities at interfaces using PRISM. Nat Protoc. 2011;6(9):1341–54. Epub 2011/09/03. 10.1038/nprot.2011.367 . 21886100 PMC7384353

[45] Faure G, Koonin EV. Universal distribution of mutational effects on protein stability, uncoupling of protein robustness from sequence evolution and distinct evolutionary modes of prokaryotic and eukaryotic proteins. Phys Biol. 2015;12(3):035001 Epub 2015/05/01. 10.1088/1478-3975/12/3/035001 25927823 PMC4770899

[46] Guo HH, Choe J, Loeb LA. Protein tolerance to random amino acid change. Proc Natl Acad Sci U S A. 2004;101(25):9205–10. Epub 2004/06/16. 10.1073/pnas.0403255101 15197260 PMC438954

[47] Lim WA, Farruggio DC, Sauer RT. Structural and energetic consequences of disruptive mutations in a protein core. Biochemistry. 1992;31(17):4324–33. Epub 1992/05/05. 10.1021/bi00132a025 . 1567879

[48] David A, Sternberg MJ. The Contribution of Missense Mutations in Core and Rim Residues of Protein-Protein Interfaces to Human Disease. J Mol Biol. 2015;427(17):2886–98. Epub 2015/07/15. 10.1016/j.jmb.2015.07.004 26173036 PMC4548493

[49] Hopf TA, Ingraham JB, Poelwijk FJ, Scharfe CP, Springer M, Sander C, et al Mutation effects predicted from sequence co-variation. Nat Biotechnol. 2017;35(2):128–35. Epub 2017/01/17. 10.1038/nbt.3769 28092658 PMC5383098

[50] Adzhubei IA, Schmidt S, Peshkin L, Ramensky VE, Gerasimova A, Bork P, et al A method and server for predicting damaging missense mutations. Nat Methods. 2010;7(4):248–9. Epub 2010/04/01. 10.1038/nmeth0410-248 ; PubMed Central PMCID: PMC2855889. 20354512 PMC2855889

[51] Chen S, Fragoza R, Klei L, Liu Y, Wang J, Roeder K, et al An interactome perturbation framework prioritizes damaging missense mutations for developmental disorders. Nat Genet. 2018;50(7):1032–40. Epub 2018/06/13. 10.1038/s41588-018-0130-z . 29892012 PMC6314957

[52] Raimondi F, Singh G, Betts MJ, Apic G, Vukotic R, Andreone P, et al Insights into cancer severity from biomolecular interaction mechanisms. Sci Rep. 2016;6:34490 Epub 2016/10/05. 10.1038/srep34490 27698488 PMC5048291

[53] Tuncbag N, Gosline SJ, Kedaigle A, Soltis AR, Gitter A, Fraenkel E. Network-Based Interpretation of Diverse High-Throughput Datasets through the Omics Integrator Software Package. PLoS Comput Biol. 2016;12(4):e1004879 Epub 2016/04/21. 10.1371/journal.pcbi.1004879 27096930 PMC4838263

[54] Wang Q, Hu B, Hu X, Kim H, Squatrito M, Scarpace L, et al Tumor Evolution of Glioma-Intrinsic Gene Expression Subtypes Associates with Immunological Changes in the Microenvironment. Cancer Cell. 2017;32(1):42–56 e6. Epub 2017/07/12. 10.1016/j.ccell.2017.06.003 28697342 PMC5599156

[55] Yang W, Soares J, Greninger P, Edelman EJ, Lightfoot H, Forbes S, et al Genomics of Drug Sensitivity in Cancer (GDSC): a resource for therapeutic biomarker discovery in cancer cells. Nucleic Acids Res. 2013;41(Database issue):D955–61. Epub 2012/11/28. 10.1093/nar/gks1111 23180760 PMC3531057

[56] van der Meer D, Barthorpe S, Yang W, Lightfoot H, Hall C, Gilbert J, et al Cell Model Passports-a hub for clinical, genetic and functional datasets of preclinical cancer models. Nucleic Acids Res. 2019;47(D1):D923–D9. Epub 2018/09/28. 10.1093/nar/gky872 30260411 PMC6324059

[57] Papadopoulos N, Lennartsson J. The PDGF/PDGFR pathway as a drug target. Mol Aspects Med. 2018;62:75–88. Epub 2017/11/16. 10.1016/j.mam.2017.11.007 . 29137923

[58] Brennan CW, Verhaak RG, McKenna A, Campos B, Noushmehr H, Salama SR, et al The somatic genomic landscape of glioblastoma. Cell. 2013;155(2):462–77. Epub 2013/10/15. 10.1016/j.cell.2013.09.034 24120142 PMC3910500

[59] UniProt Consortium T. UniProt: the universal protein knowledgebase. Nucleic Acids Res. 2018;46(5):2699 Epub 2018/02/10. 10.1093/nar/gky092 29425356 PMC5861450

[60] Zerbino DR, Achuthan P, Akanni W, Amode MR, Barrell D, Bhai J, et al Ensembl 2018. Nucleic Acids Res. 2018;46(D1):D754–D61. Epub 2017/11/21. 10.1093/nar/gkx1098 29155950 PMC5753206

[61] Mitternacht S. FreeSASA: An open source C library for solvent accessible surface area calculations. F1000Res. 2016;5:189 Epub 2016/03/15. 10.12688/f1000research.7931.1 26973785 PMC4776673

[62] Turner B, Razick S, Turinsky AL, Vlasblom J, Crowdy EK, Cho E, et al iRefWeb: interactive analysis of consolidated protein interaction data and their supporting evidence. Database (Oxford). 2010;2010:baq023 Epub 2010/10/14. 10.1093/database/baq023 20940177 PMC2963317

[63] Hristov BH, Singh M. Network-Based Coverage of Mutational Profiles Reveals Cancer Genes. Cell Syst. 2017;5(3):221–9 e4. Epub 2017/09/29. 10.1016/j.cels.2017.09.003 28957656 PMC5997485

[64] Wang J, Vasaikar S, Shi Z, Greer M, Zhang B. WebGestalt 2017: a more comprehensive, powerful, flexible and interactive gene set enrichment analysis toolkit. Nucleic Acids Res. 2017;45(W1):W130–W7. Epub 2017/05/05. 10.1093/nar/gkx356 28472511 PMC5570149

[65] Huang JK, Jia T, Carlin DE, Ideker T. pyNBS: a Python implementation for network-based stratification of tumor mutations. Bioinformatics. 2018;34(16):2859–61. Epub 2018/04/03. 10.1093/bioinformatics/bty186 29608663 PMC6084608

