# Supplementary figures and images for: 3D spatial organization and network-guided comparison of mutation profiles in Glioblastoma reveals similarities across patients
Source: PLoS Comput Biol. 2019 Sep 17;15(9):e1006789. doi: 10.1371/journal.pcbi.1006789 (PMC6782092; doi:10.1371/journal.pcbi.1006789)

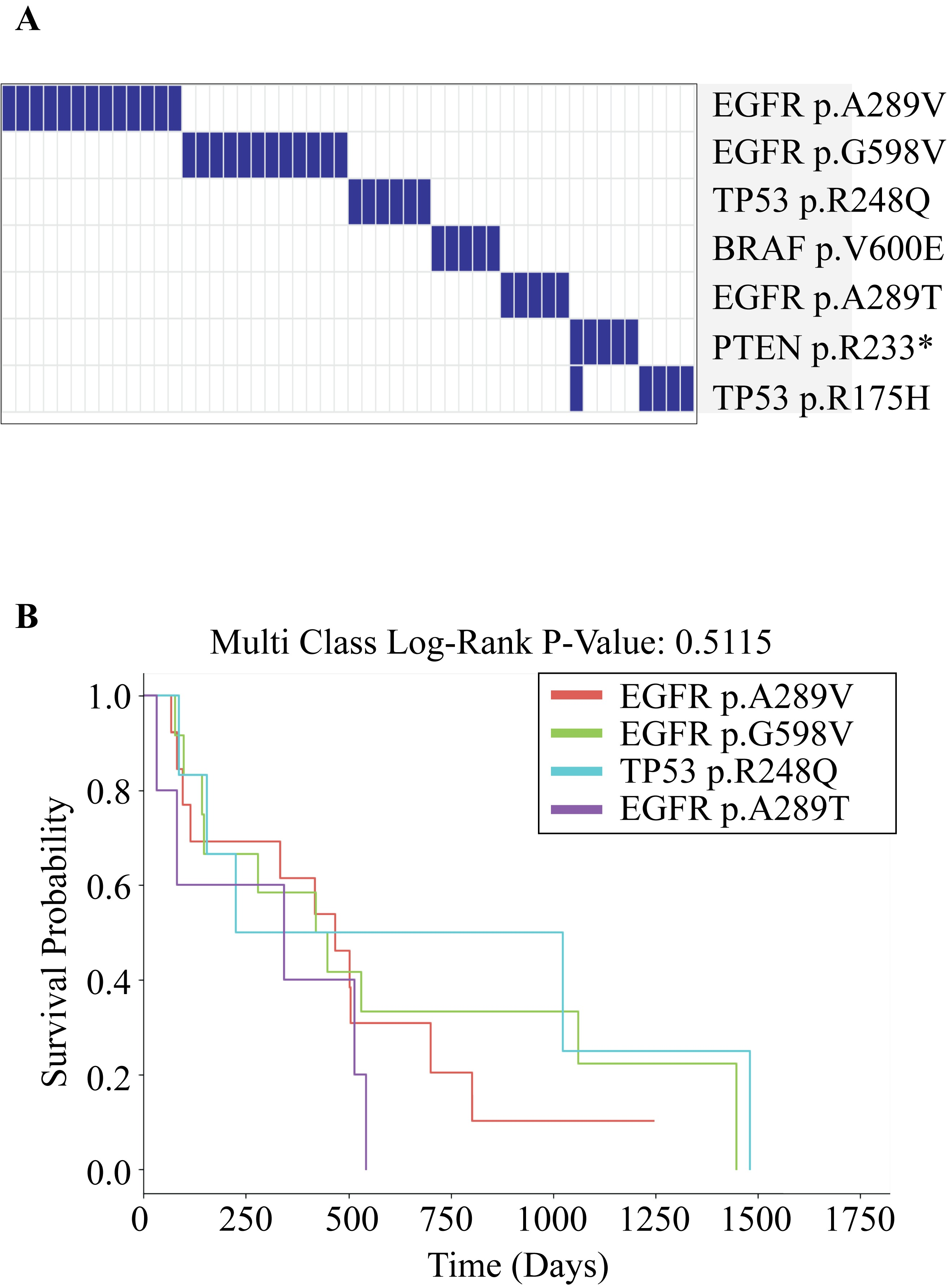

Supplement: S1 Fig — (A) Grouping patients based on individual mutations. Each column represents a patient and each row represents a mutation. (B) Association between Kaplan-Meier survival curves and patient groups by the most frequent individual mutations. (TIF) [file pcbi.1006789.s002.tif]

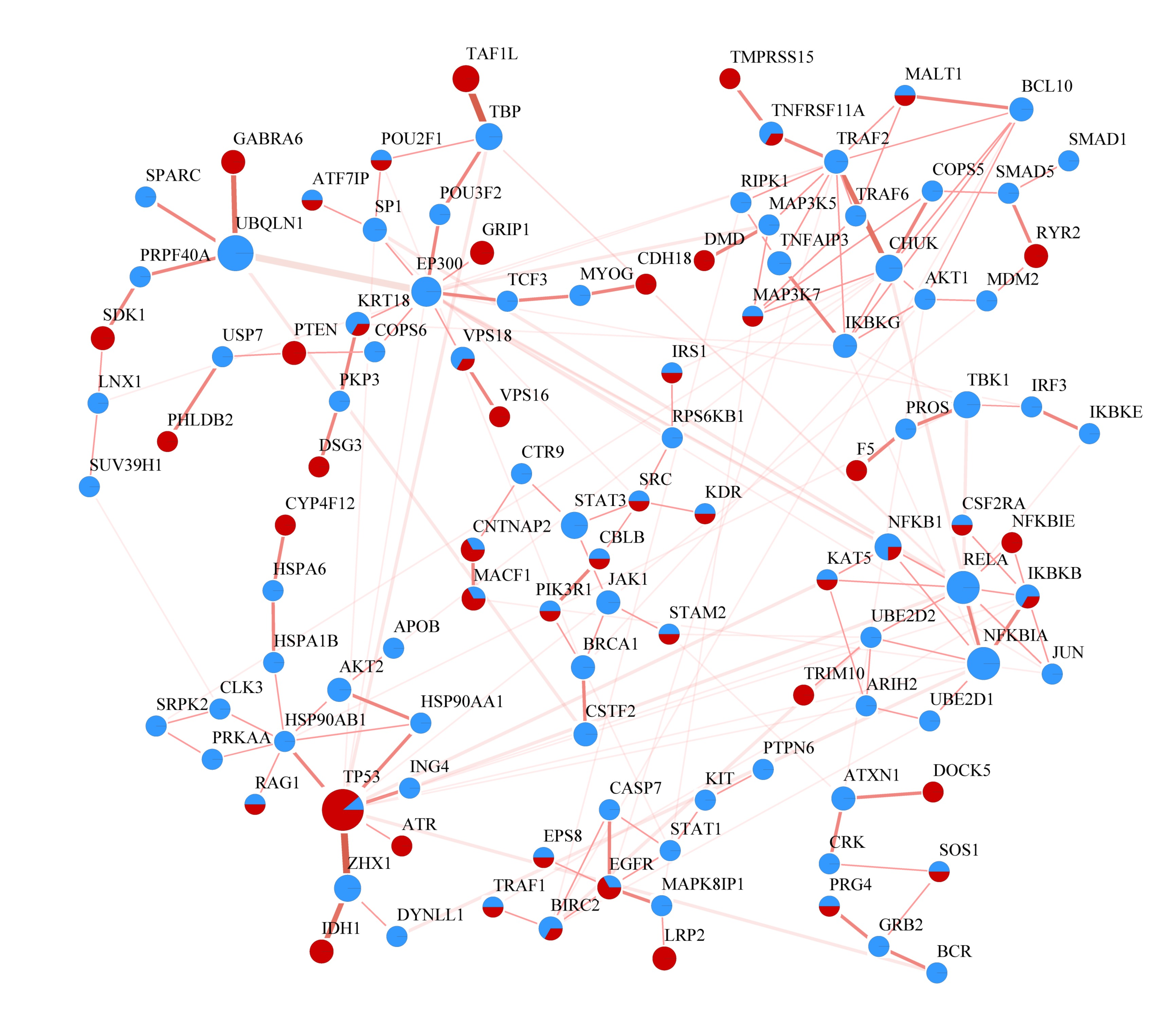

Supplement: S2 Fig — The nodes are labelled with a pie chart colored in red and/or blue color. The fraction of the red color represents the count of being a mutated protein in the patient network. The fraction of the blue color represents the count of being an intermediate protein connecting mutated ones in the patient network. Cytoscape is used for network visualization. (TIF) [file pcbi.1006789.s003.tif]

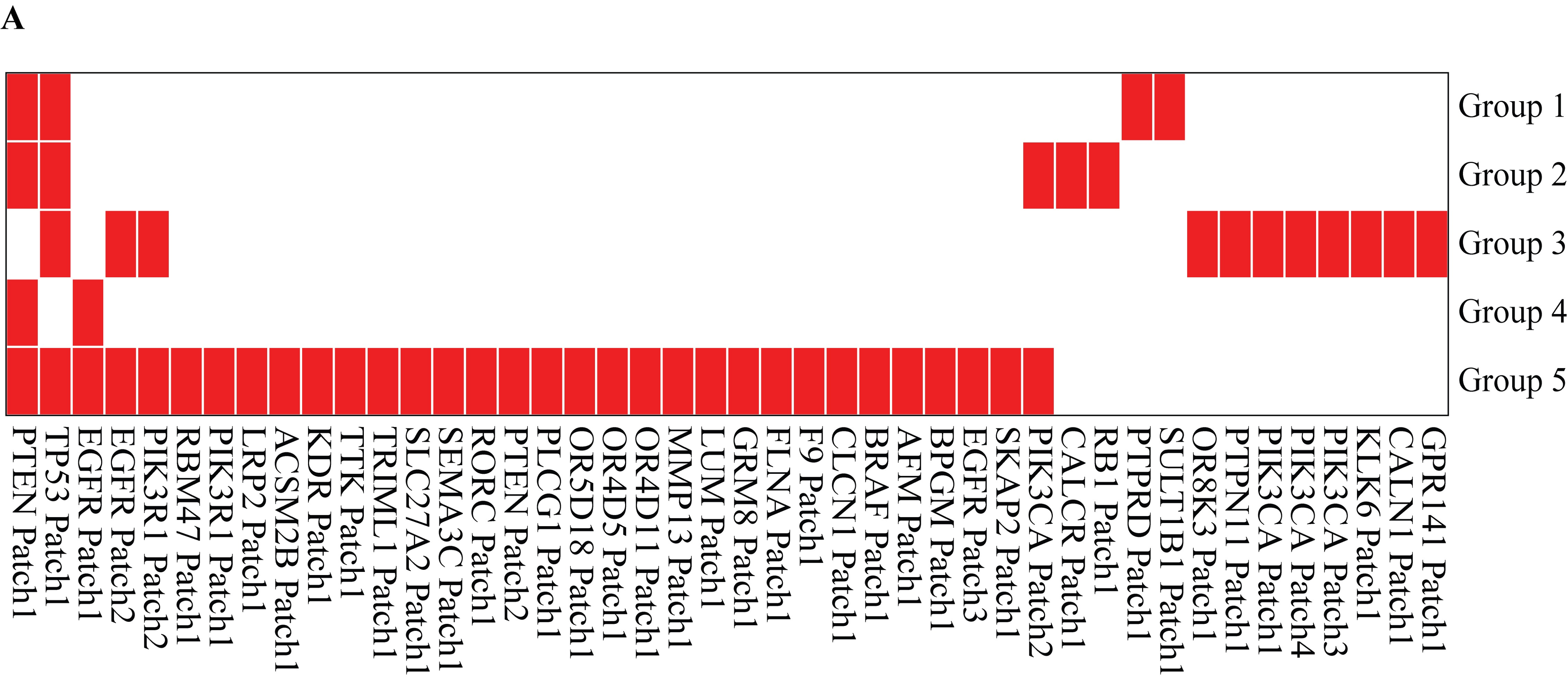

Supplement: S3 Fig — Columns are patches and rows are patient groups. Red color represents the presence of the corresponding patch in the patient group. (TIF) [file pcbi.1006789.s004.tif]

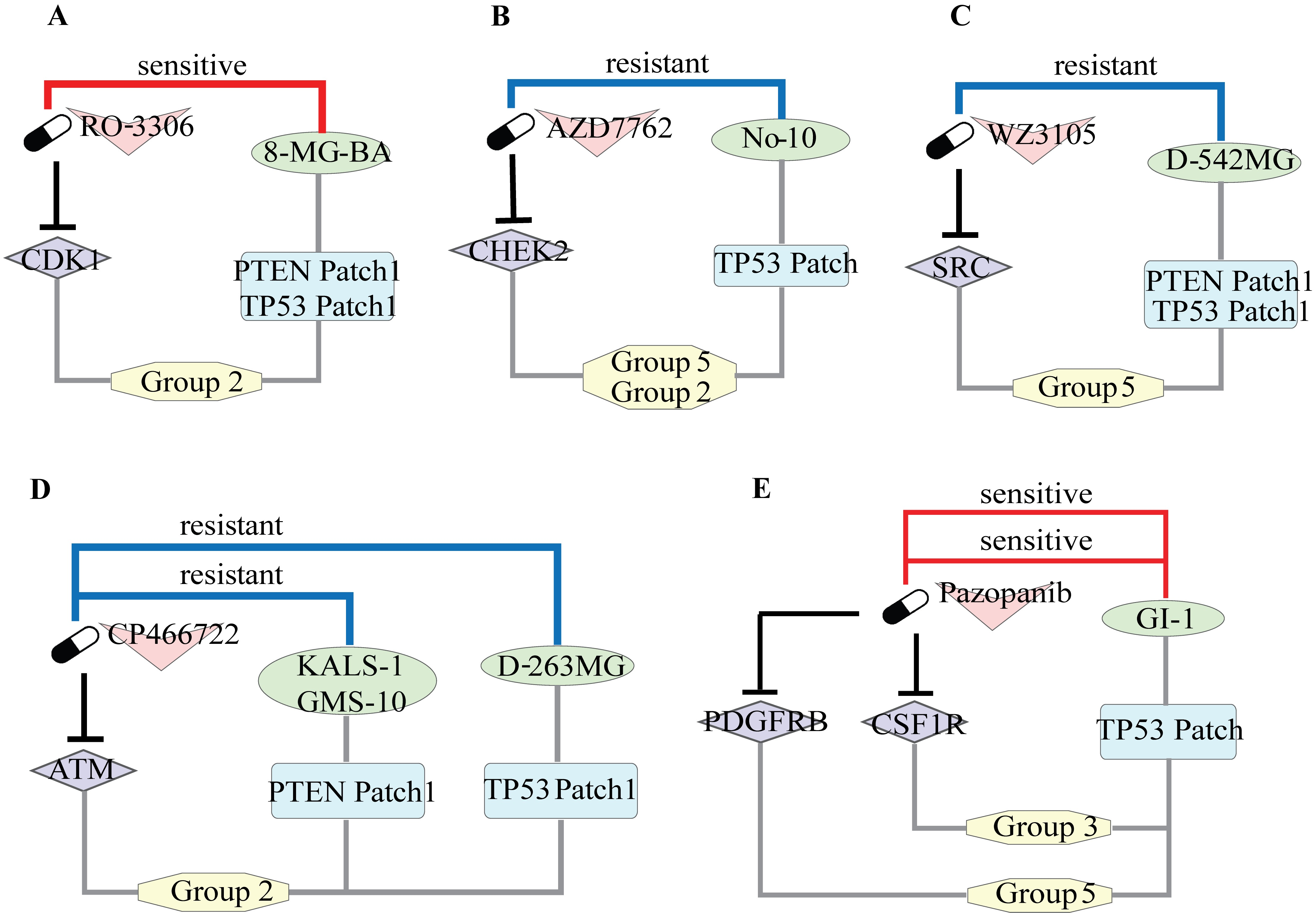

Supplement: S4 Fig — (A, B) Additional therapeutic hypotheses where the first one is RO-3306 (targeting CDK1) for Group 2, the second is the possible resistance of Group 2 and Group 5 to CHEK2 inhibition by AZD7762. (TIF) [file pcbi.1006789.s005.tif]
